# DNMT3L Is a Regulator of X Chromosome Compaction and Post-Meiotic Gene Transcription

**DOI:** 10.1371/journal.pone.0018276

**Published:** 2011-03-31

**Authors:** Natasha M. Zamudio, Hamish S. Scott, Katja Wolski, Chi-Yi Lo, Charity Law, Dillon Leong, Sarah A. Kinkel, Suyinn Chong, Damien Jolley, Gordon K. Smyth, David de Kretser, Emma Whitelaw, Moira K. O'Bryan

**Affiliations:** 1 The Department of Anatomy and Developmental Biology, Monash University, Victoria, Australia; 2 The Australian Research Council Centre of Excellence in Biotechnology and Development, Monash University, Victoria, Australia; 3 The Monash Institute of Health Services Research, Monash University, Victoria, Australia; 4 The Institute of Medical and Veterinary Science, University of Adelaide, Adelaide, Australia; 5 The Walter and Eliza Hall Institute of Medical Research, Parkville, Victoria, Australia; 6 Queensland Institute of Medical Research, Herston, Queensland, Australia; 7 Department of Medical Biology, University of Melbourne, Victoria, Australia; University of Southern California, United States of America

## Abstract

Previous studies on the epigenetic regulator DNA methyltransferase 3-Like (DNMT3L), have demonstrated it is an essential regulator of paternal imprinting and early male meiosis. *Dnmt3L* is also a paternal effect gene, i.e., wild type offspring of heterozygous mutant sires display abnormal phenotypes suggesting the inheritance of aberrant epigenetic marks on the paternal chromosomes. In order to reveal the mechanisms underlying these paternal effects, we have assessed X chromosome meiotic compaction, XY chromosome aneuploidy rates and global transcription in meiotic and haploid germ cells from male mice heterozygous for *Dnmt3L*. XY bodies from *Dnmt3L* heterozygous males were significantly longer than those from wild types, and were associated with a three-fold increase in XY bearing sperm. Loss of a *Dnmt3L* allele resulted in deregulated expression of a large number of both X-linked and autosomal genes within meiotic cells, but more prominently in haploid germ cells. Data demonstrate that similar to embryonic stem cells, DNMT3L is involved in an auto-regulatory loop in germ cells wherein the loss of a *Dnmt3L* allele resulted in increased transcription from the remaining wild type allele. In contrast, however, within round spermatids, this auto-regulatory loop incorporated the alternative non-coding alternative transcripts. Consistent with the mRNA data, we have localized DNMT3L within spermatids and sperm and shown that the loss of a *Dnmt3L* allele results in a decreased DNMT3L content within sperm. These data demonstrate previously unrecognised roles for DNMT3L in late meiosis and in the transcriptional regulation of meiotic and post-meiotic germ cells. These data provide a potential mechanism for some cases of human Klinefelter's and Turner's syndromes.

## Introduction

Infertility affects 1 in 20 men in the Western world. Increasingly data is showing that in addition to genetic changes, aberrant epigenetic regulation during spermatogenesis can lead to human infertility [1]–[3]. Previous studies on the epigenetic regulator DNMT3L, have shown that deletion of *Dnmt3L* in mice resulted in hypomethylation of paternally imprinted regions and transposable elements in gonocytes, as well as developmental arrest and death of early meiotic cells [4]–[6]. Consequently *Dnmt3L* knockout males were sterile.

*Dnmt3L* mRNA and protein were originally reported only in the primordial germ cells, where DNMT3L is needed to establish epigenetic marks required in early primary spermatocytes [4], [7]. Subsequently, it was shown that *Dnmt3L* mRNA was present in spermatocytes and spermatids [5], [8]. Furthermore, it was shown that a second promoter in intron 9 initiates three alternative transcripts (Forms 1, 2 and 3) in late pachytene spermatocytes and round spermatids [9]. While the function of the alternative isoforms is unknown, they are thought to be non-coding and to suppress translation of the full length *Dnmt3L* isoform [9]. The localization of DNMT3L protein within the adult testis or sperm has not been reported.

*Dnmt3L* was recently reported as the first example of a mammalian paternal effect gene [10]. Animals with mutations in such genes are of particular interest as they exhibit a male heterozygous phenotype, that can affect the phenotype of their wild type progeny. Specifically, when *Dnmt3L* heterozygous males were mated to wild type females, there was an increased incidence of XO monosomy in wild type E10.5 embryos and adults compared to wild type matings [10]. These data suggested that the *Dnmt3L* heterozygous males may have a meiotic defect related to X and Y chromosome segregation and encouraged us to study the effects of the loss of a *Dnmt3L* allele in germ cells in more detail.

We hypothesised that decreased availability of DNMT3L in *Dnmt3L* heterozygous males may lead to the inappropriate epigenetic marking of the DNA, and a partial failure of sex chromosomes to convert from an euchromatic state to a highly condensed heterochromatic state leading to a meiotic instability. We hypothesised that if true, abnormal XY compaction would be reflected by aberrant patterns of gene expression in meiotic and haploid germ cells. To investigate these hypotheses we measured the lengths of the XY bodies in meiotic cells from heterozygous and wild type males, we defined the incidence of XY sperm aneuploidies, and monitored transcriptional activity in spermatocytes and round spermatids.

*Dnmt3L* heterozygous males had XY bodies which were significantly longer than those from their wild type brothers, and exhibited a significantly increased incidence of XY non-disjunction. We demonstrated that a loss of a *Dnmt3L* allele was associated with disregulation of a number of X-linked and autosomal genes in spermatocytes and spermatids. Consistent with these observations, we localized DNMT3L protein within spermatids and mature sperm. Furthermore, our data strongly suggested a complex *Dnmt3L* self-regulatory mechanism in germ cells similar to that previously reported in embryos and ES cells [11], but additionally involving the alternative transcripts.

## Results

### *Dnmt3L* heterozygous males displayed XY body abnormalities

**Figure 1:**
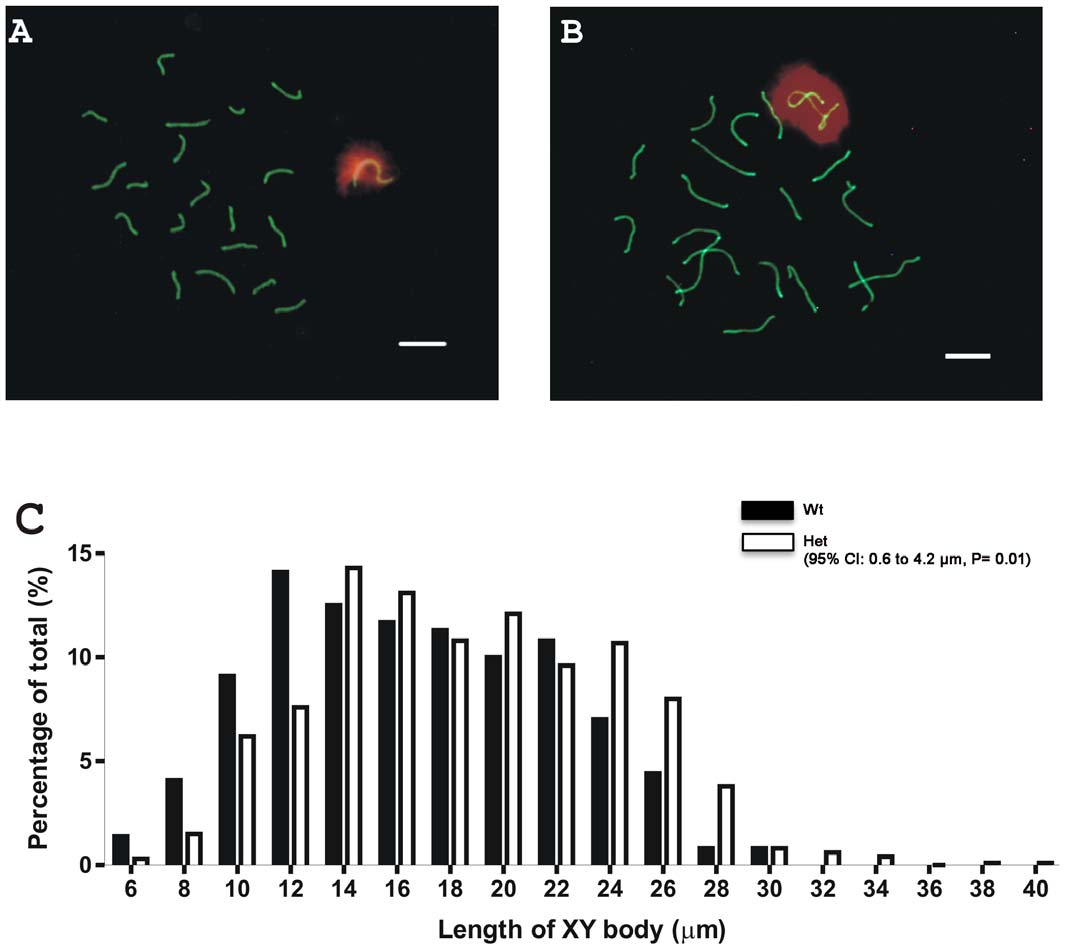
*Dnmt3L* haploinsufficiency resulted in XY body abnormalities. (**A**) A typical pachytene spermatocyte from a *Dnmt3L* wildtype male. (**B**) A pachytene spermatocyte from a *Dnmt3L* heterozygous male. The XY body appeared to be twisted and longer when compared to the XY body from wildtype males. Green  =  SCP3, red  =  γH2AX. Scale bars  = 10 µm. (**C**) Lengths of XY bodies from *Dnmt3L* heterozygous (white bars, n = 712 cells, n = 8 mice) and wildtype males (black bars, n = 650 cells, n = 6 mice).

During male meiosis, the X and Y chromosomes form an XY body and become transcriptionally silent in a process known as meiotic sex chromosome inactivation (MSCI) [12], [13]. Unlike the autosomes, in males sex chromosomes are heterologous and only synapse at their pseudoautosomal regions. To test the hypothesis that DNMT3L has a role in X chromosome meiotic condensation, we measured the lengths of the XY bodies in *Dnmt3L* ^(−/+)^ and *Dnmt3L* ^(+/+)^ males. The X and Y chromosomes typically join at their pseudoautosomal regions to form a highly condensed and inactive XY body during the pachytene stage of meiosis (Fig. 1A). Many studies have conducted meiotic spreads in order to look for the localization of proteins on the XY body during pachytene [14], [15], however, to our knowledge there have not been any studies on the physical conformation of the XY body. As such this study necessitated defining the normal spectrum of XY lengths within pachytene spermatocytes from wild type mice.

To assess XY body length in a quantitative and unbiased manner we measured the lengths of XY bodies in meiotic spreads from wild type and *Dnmt3L* heterozygous males. All measurements were conducted without knowledge of genotype. For each group (C57Bl/6 wild type, *Dnmt3L* ^(+/+)^ and *Dnmt3L^(^* ^−/+)^) the percentage of XY bodies of a particular length was determined (Fig. 1C). Phosphorylated Histone 2AX (γH2AX) was used as an XY body marker. Synaptonemal complex protein 3 (SCP3) was used to label chromosome pairs. In order to assess the possibility that *Dnmt3L* wild type males may also have a meiotic defect we included a C57Bl/6 control mouse group in addition to wild type males from the *Dnmt3L* colony.

Consistent with previously published data showing that the degree of XY body compaction increases throughout pachytene [16], meiotic spreads from wild type males displayed a range of lengths spanning 6 to 30 µm (Fig. 1C). Compared to meiotic spreads from wild type animals, the XY bodies from heterozygous males appeared abnormal and longer more often than those from wild type males (Fig. 1A and B). A random-effects general linear model found that the mean length for the *Dnmt3L* heterozygotes XY bodies was 2.4 µm longer than the mean length for the *Dnmt3L* wild type XY bodies (95% CI: 0.6 to 4.2 µm, P = 0.01). It is evident that the *Dnmt3L* wild type males had a larger percentage of XY bodies with shorter lengths when compared to the *Dnmt3L* heterozygotes, (Fig. 1C). No significant difference was observed between the lengths and distribution of the *Dnmt3L* wild type and the C57Bl/6 wild type XY bodies (95% CI: −2.6 to +1.1, P = 0.42) (data not shown). These data suggest that the loss of a *Dnmt3L* allele resulted in a partial failure of XY body chromatin compaction during meiosis. Interestingly, we observed no abnormalities in autosome pairing in germ cells from *Dnmt3L* heterozygous males (data not shown).

### *Dnmt3L* heterozygous males had an increased risk of producing XY sperm

**Figure 2:**
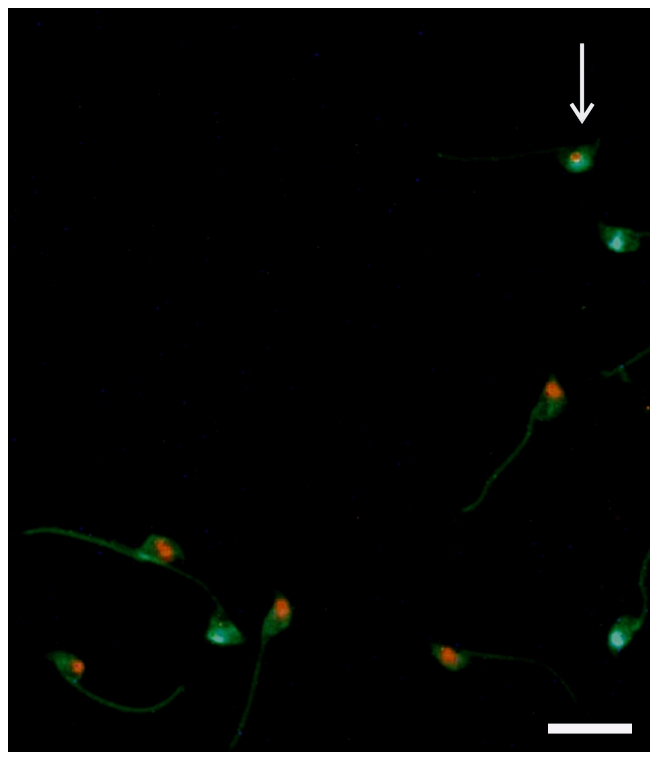
*Dnmt3L* haploinsufficiency resulted in an increased risk of XY sperm. FISH on sperm from a *Dnmt3L* heterozygous male with X and Y chromosome probes. Green (FITC)  =  X chromosome, Red (CY3)  =  Y chromosome. Arrow indicates an XY spermatozoon. Scale bar  =  10 µm.

To address the hypothesis that a partial failure of XY body compaction would lead to an increase in XY bearing sperm in *Dnmt3L* heterozygous males, we used chromosome paints to label decondensed sperm for X and Y chromosomes (Fig. 2). The visual assessments and counting were all conducted without knowledge of genotype. Sperm with no labelling were not included in the analysis in order to avoid the possibility of false negative sperm as a consequence of incomplete sperm head decondensation. The number of sperm lacking a sex chromosome should however, equal the number carrying both an X and a Y chromosome.

From a total of 2,063 sperm (from 9 mice) we found 7 sperm with a XY chromosome content in wild type males, compared to 22 sperm (from 8 mice) from a total of 2,155 sperm in *Dnmt3L* heterozygous males (Supporting Information File S1). The total number of X and/or Y bearing sperm from wild type and *Dnmt3L* heterozygous males (analysed separately) was the same, suggesting that the FISH probes worked equally well and that the loss of a *Dnmt3L* allele did not result in a sex chromosome bias at the level of the sperm (data not shown). A negative binomial regression analysis showed the heterozygous males had three times the risk of sperm having a XY content, compared to sperm from wild type males (P = 0.024). As *Dnmt3L* wild type and heterozygous males had equivalent rates of daily sperm production (Supporting Information File S1), this meant that *Dnmt3L* heterozygous males produced three times more XY bearing sperm. In humans XY sperm leads to the most common chromosome aneuploidy, Klinefelter's syndrome which occurs with a frequency of greater than 1 in 1000 male live births [17], [18].

An analysis using a probe against chromosome 16 (the equivalent to human chromosome 21), did not show a difference in the frequency of aneuploid sperm from *Dnmt3L* heterozygous males compared to wild type males (data not shown). This result fits in with our observation of no observable increase in autosomal pairing defects during meiosis.

### A number of genes were differentially expressed in *Dnmt3L* heterozygous males

As an extension of the incomplete XY chromatin condensation, we hypothesised that *Dnmt3L* heterozygosity would lead to the inappropriate transcription of genes in meiotic and post-meiotic germ cells. To test this, we conducted microarray analyses on mRNA from purified primary spermatocyte and spermatid populations from wild type and heterozygous males. Cells were purified from 10 animals per replicate, and three biological replicates (30 animals) were assessed. About 60% of the Illumina probes (27,994 probes) were found to be expressed in primary spermatocytes or spermatids. Of these, fifty three were differentially expressed in spermatocytes between *Dnmt3L* wild type and heterozygous males at a 5% false discovery rate (FDR) (Supporting Information File S1). Twenty nine probes (27 unique genes) were up-regulated and twenty four probes (23 genes) were down-regulated in *Dnmt3L* heterozygous spermatocytes compared to spermatocytes from wild types. Within spermatids, one hundred and twenty three probes were differentially expressed between *Dnmt3L* wild type and heterozygous animals (FDR <0.05) (Supporting Information File S1). Of these, seventy five probes (73 genes) were up-regulated and forty eight probes (46 genes) were down-regulated in *Dnmt3L* heterozygous spermatids compared to the wild type. The raw data from our microarray analyses have been submitted to GenBank GEO.

When we restricted the analysis of the microarray data to the 855 X chromosome probes, twelve genes were differentially expressed between spermatocytes from *Dnmt3L* wild type and heterozygous males, at FDR <0.20 (Supporting Information File S1) and 57 genes were differentially expressed between *Dnmt3L* wild type and heterozygous spermatids at FDR <0.20 (Supporting Information File S1). An MA-plot showed a trend whereby X-linked genes which were relatively highly expressed in spermatocytes tended to be over-expressed in *Dnmt3L* heterozygous males (data not shown). In contrast, all the differentially expressed X chromosome genes in spermatids were down-regulated in *Dnmt3L* heterozygous males.

**Figure 3:**
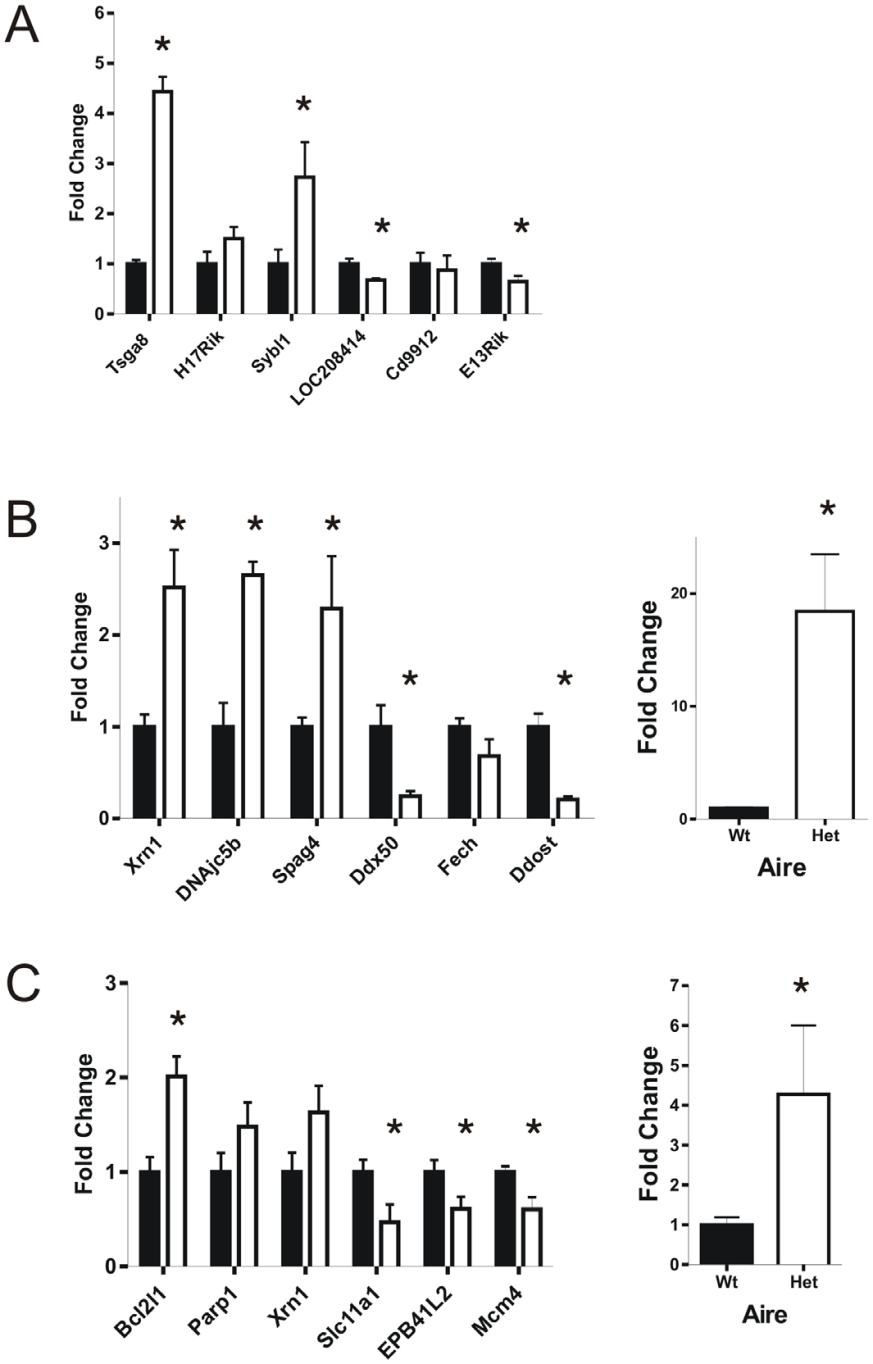
QPCR on selected genes differentially expressed in *Dnmt3L* wildtype versus *Dnmt3L* heterozygous germ cells by microarray. (**A**) X-linked genes from spermatocytes. (**B**) Autosomal genes from spermatocytes. (**C**) Autosomal genes in spermatids. Expression was normalized to β-actin and the wildtype values were set to 1. Black bars  =  wildtype, white bars  =  heterozygous, n = 3 biological replicates (10 mice/replicate). Results are presented as means ± SEM. * Indicates a statistically significant increase or decrease in gene expression with a p≤0.05.

To independently assess the microarray data, we conducted QPCR on RNA from purified spermatocytes and spermatids. We examined 6 X-linked genes and 7 autosomal genes that were differentially expressed in spermatocytes (Fig. 3A and 3B), and 7 autosomal genes that were differentially expressed in spermatids (Fig. 3C). Genes were selected based on their relative change in expression as found in the microarray data and their previously recognized roles in spermatogenesis. As for the microarrays, cells were purified from 10 males per biological replicate and a minimum of 3 biological replicates were assessed per experiment. QPCR verified all of the changes in gene expression detected by microarray (Fig. 3).

Collectively, the microarray and QPCR data showed that *Dnmt3L* heterozygosity resulted in significant changes in spermatocyte and spermatid autosomal gene transcription. Altered spermatocyte X chromosome gene expression was also evident, however, these changes were relatively few in number and often low in magnitude. This data was supported by RNA polymerase II staining of the XY body (as a marker of transcriptional activity) (Supporting Information File S1), which showed that the XY bodies from *Dnmt3L* heterozygous males contained on average 10% more RNA polymerase II labelling than XY bodies from wild type males. This data supports a role for DNMT3L in the regulation of autosomal gene transcription in spermatocytes and round spermatids.

### Deregulated *Dnmt3L* expression in germ cells from *Dnmt3L* heterozygous males

**Figure 4:**
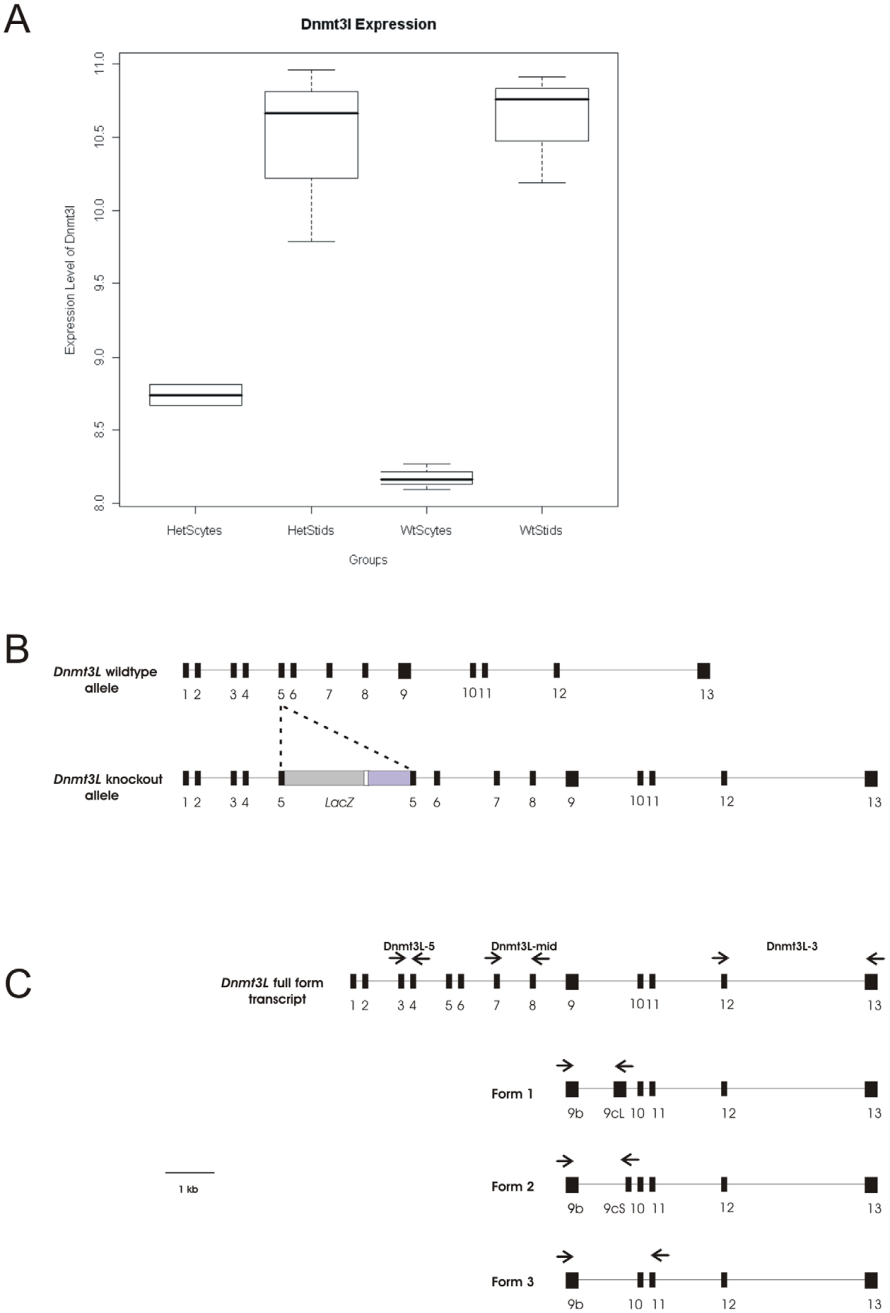
*Dnmt3L* expression in *Dnmt3L* haploinsufficient males. (**A**) A box plot representing the total *Dnmt3L* expression from microarray on *Dnmt3L* heterozygous and wildtype spermatocytes and spermatids. The expression of *Dnmt3L* was normalised to the whole genome, n = 3 biological replicates (10 mice per replicate). (**B**) The *Dnmt3L* wildtype allele contains 13 exons. Disruption of *Dnmt3L* was achieved through homologous recombination and resulted in the insertion of a *LacZ* gene within exon 5. Three stop codons and a polyadenylation signal were included after the *LacZ* gene. (**C**) The Dnmt3L-5 primer sets were located in exons 3–4, 5′ to the insertion of the *LacZ* gene. The Dnmt3L-mid primer sets were located in exons 7–8, 3′ of the insertion of the *LacZ* gene. The Dnmt3L-3 primer sets were located in exons 12–13, which is common to all four *Dnmt3L* isoforms. A second promoter can be activated in intron 9 of the *Dnmt3L* gene to produce a novel exon, 9b, from which transcription of the three alternative transcripts starts. Form 1 includes exon 9b and large 9c (9cL). Form 2 includes exon 9b and short 9c (9cS). Form 3 only includes exon 9b. Forms 1, 2 and 3 all contain exons 10–13. Primers for Form 1 were located in exons 9b and 9cL. Primers for Form 2 were located in exons 9b and 9cS. Primers for Form 3 were located in exon 9b and exon 11. All primer sets are represented by arrows. (**A, B**) Black boxes represent exons. Thin lines between exons represent introns.

In addition to measuring thousands of other genes, the microarrays assessed *Dnmt3L* expression. Consistent with the data of Hata *et al* and La Salle *et al* [5], [8], our data clearly showed that *Dnmt3L* was expressed in wild type spermatocytes and spermatids (Fig. 4A). It also showed that spermatids have a relatively higher *Dnmt3L* mRNA content than spermatocytes (Fig. 4A).

Contrary to our expectations, germ cells (both pachytene spermatocytes and round spermatids) from heterozygous males did not have a decreased level of *Dnmt3L* mRNA compared to those from wild type animals, as determined using a probe complementary to exon 13 (Fig. 4A). Heterozygous males carry both a *Dnmt3L* knockout and a wild type allele (Fig. 4B). It is of note, that male germ cells develop within a syncitium wherein mRNA and proteins produced in one cell can be shared across cytoplasmic bridges, meaning that although individual spermatids may have a different genotype i.e. heterozygous or wild type for *Dnmt3L*, they are equivalent at a protein and mRNA level [19]. The analysis done herein also showed that the expression of *Dnmt3L* was not significantly different between pachytene spermatocytes and round spermatids from *Dnmt3L* heterozygous and wild type males (Fig. 4A). In light of the observed meiosis and transcription phenotype, this led us to investigate the relative expression of each of the four *Dnmt3L* transcripts in spermatocytes and spermatids from *Dnmt3L* heterozygous and wild type.

Exon 13 is contained within the *Dnmt3L* full protein coding mRNA and all of the alternative transcripts. Forms 1, 2 and 3 transcripts start with an alternative exon 9, designated exon 9b (Fig. 4C) [9]. Form 1 contains exon 9b and a second novel exon, exon 9cL. Form 2 contains exon 9b and a shorter version of exon 9c, exon 9cS. Form 3 contains exon 9b only. All three forms contain the same exons 10, 11, 12 and 13 as in the *Dnmt3L* full form mRNA, and were therefore detected on the microarray.

In order to assess the expression levels of individual *Dnmt3L* isoforms in spermatocytes and spermatids, we designed QPCR assays specific for the three alternative transcripts as well as assays to detect the 5′, middle and 3′ regions of the *Dnmt3L* full form transcript (Fig. 4C, Supporting Information File S1). The primers used for Form 1, Form 2 and Form 3 were as used by Shovlin *et al* (Supporting Information File S1) [9]. Based on previously published data, only mRNA transcribed from the *Dnmt3L* full form transcript results in functional protein [9]. While the mRNA for Forms 1, 2 and 3 isoforms share significant homology with the protein coding splice variant at the level of mRNA sequence, they do not contain an open reading frame of any length and as such can not produce protein. Relative expression was measured in a minimum of three biological replicates and each replicate contained purified germ cells from 10 males.

**Figure 5:**
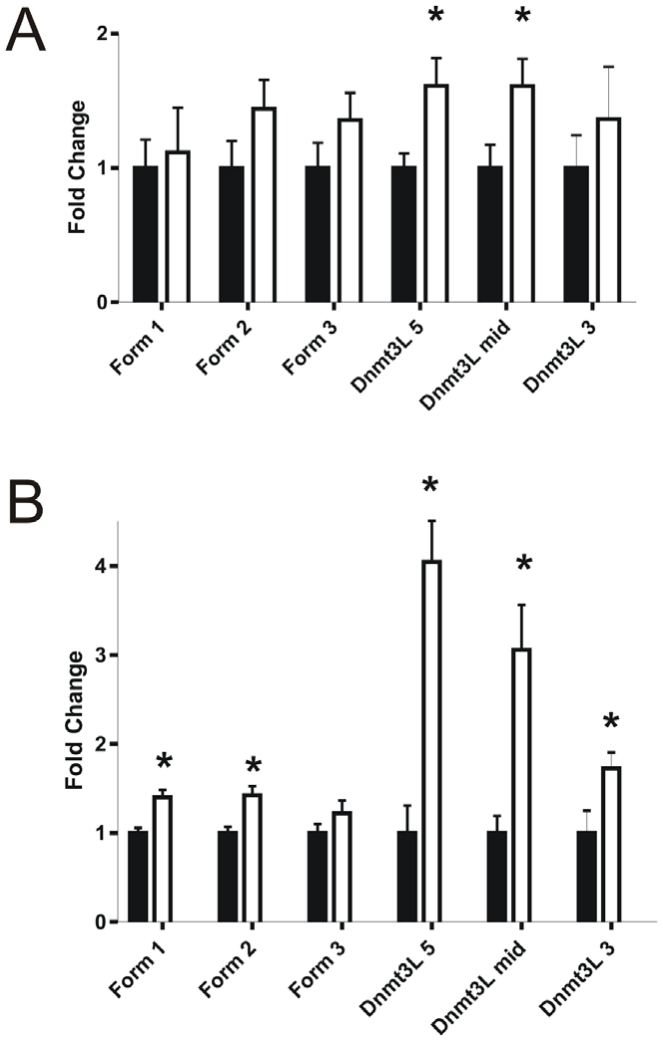
*Dnmt3L* haploinsufficiency resulted in altered expression of the *Dnmt3L* splice variants. The level of *Dnmt3L* mRNA transcripts in spermatocytes (**A**) and spermatids (**B**) from *Dnmt3L* heterozygous and wildtype males as defined by QPCR. Each gene was normalized to β-actin and the wildtype values were set to 1. Black bars  =  wildtype, white bars  =  heterozygous, n = 3 biological replicates (10 mice/replicate). Results are presented as means ± SEM. * Indicates a statistically significant increase or decrease in gene expression with a p≤0.05.

QPCR on spermatocytes from *Dnmt3L* heterozygous and wild type males showed that the loss of a *Dnmt3L* allele resulted in a significant up-regulation of the 5′ (P = 0.0223) and mid (P = 0.0417) regions of the full *Dnmt3L* transcripts in the heterozygous males (Fig. 5A). While the up-regulation in mRNA containing the 5′ sequence could be from either the wild type or knockout allele, the up-regulation in mRNA containing the Dnmt3L-mid sequence suggested an up-regulation from the wild type allele i.e. translation from the knockout allele would be blocked by the stop codons in the *LacZ* cassette (Fig. 4B). The expression of Form 1, 2, 3 and the Dnmt3L-3 products was comparable between spermatocytes from *Dnmt3L* heterozygous and wild type males (Fig. 5A).

Similarly, QPCR on spermatids from *Dnmt3L* heterozygous and wild type males showed a significant up-regulation of the 5′ (P = 0.0027), mid (P = 0.0094) and 3′ (P = 0.0391) regions of the full *Dnmt3L* transcript in the heterozygous males (Fig. 5B). In addition, there was a significant increase in the expression of Form 1 (P = 0.008) and 2 (P = 0.0129) transcripts in *Dnmt3L* heterozygous spermatids (Fig. 5B).

Collectively, these data showed that in addition to a compensatory up-regulation of the *Dnmt3L* full length transcript within spermatocytes and spermatids from *Dnmt3L* heterozygous males, there was an up-regulation in the use of the 3′ promoter and the transcription of the alternative non-coding *Dnmt3L* forms, Forms 1 and 2, within spermatids. The up-regulation of the *Dnmt3L* isoforms suggests that the *Dnmt3L* knockout reported by Webster *et al* did not result in full ablation of the gene (Fig. 4B).

### *DNMT3L* is found in the adult testis and sperm

**Figure 6:**
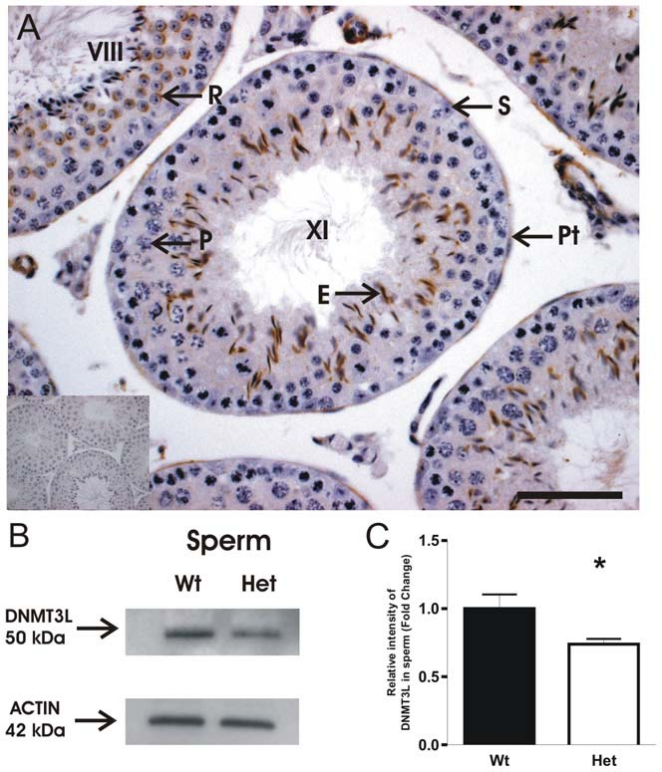
DNMT3L was found within the adult testis and mature sperm. (**A**) DNMT3L was localized to the nuclei of round spermatids (R), elongating spermatids (E) and peritubular cells (Pt). The omission of primary antibody was used as a negative control (inset). S  =  spermatogonia, P  =  pachytene spermatocyte. Scale bar  = 50 µm. (**B**) Western Blot analysis of DNMT3L (50 kDa) within *Dnmt3L* wildtype and heterozygous sperm. (**C**) Relative content of DNMT3L within sperm. Each sample was normalized to Actin and the wildtype value was set to 1. Black bars  =  wildtype, white bars  =  heterozygous, n = 3 biological replicates (2 mice/replicate). Results are presented as means ± SEM. * Indicates a statistically significant decrease in relative intensity of DNMT3L with a p≤0.05.

Our microarray and QPCR data showed that splice variants of *Dnmt3L* were present in spermatocytes and spermatids, consistent with previously published data [8]. To assess the possibility that the *Dnmt3L* full length transcript was translated, we determined the localization of DNMT3L in testis sections using immunohistochemistry with antibodies directed against both the N-terminal and C-terminal of the full length isoform. Using both antibodies we detected DNMT3L in the nuclei of round spermatids, the heads of the elongating spermatids and in peritubular cells (Fig. 6A). This is the first report of DNMT3L in the adult testis.

The presence of DNMT3L in sperm from *Dnmt3L* wild type and heterozygous males was conclusively shown by Western blotting (Fig. 6B). Further, and consistent with the *Dnmt3L* isoform data described above, the relative content of DNMT3L in sperm from *Dnmt3L* heterozygous males was reduced by 28% compared to sperm from wild type males (P = 0.027, n = 3 biological replicates with 2 mice per replicate) (Fig. 6C).

## Discussion

These data show for the first time, that DNMT3L is a regulator of both XY body compaction and ultimately XY chromosomal segregation. The XY bodies from *Dnmt3L* heterozygous males are longer than those from *Dnmt3L* and C57Bl/6 wild type males, and this finding supports a mechanism whereby a partial failure of chromatin compaction in the *Dnmt3L* heterozygous males leads to paternal effects [10]. Further, our data reveals that loss of a *Dnmt3L* allele results in dysregulation of both meiotic and post-meiotic gene transcription.

It is established that much of the XY body becomes condensed and silenced during pachytene [12]–[14]. Our results suggest that in the heterozygous males, the chromatin of the XY body is not condensing as tightly as in the wild type males. As a result of this, some genes that should become inactived may remain accessible for transcription. Our data shows that DNMT3L is involved in regulating some of the chromatin changes occurring on the XY body. These changes may be important for the silencing of certain genes.

Previous studies have shown that DNMT3L is essential during the early phases of male meiosis. The absence of *Dnmt3L* results in a failure to silence retrotransposon expression in gonocytes, and a failure of homologous chromosome condensation and pairing in leptotene and zygotene spermatocytes [4], [6]. The subsequent “meiotic catastrophe” is a consequence of a failure of chromosomes to progress from a euchromatic state to a heterochromatic state [6]. Recently it was also shown that in addition to a failure of synapsis during meiosis, *Dnmt3L* null males have an impairment of the meiotic silencing by unsynapsed chromatin (MSUC) response. As such this resulted in a failure of XY body silencing which may contribute to the *Dnmt3L* mutant phenotype [20]. The data presented here suggest that a similar, but more subtle, mechanism is occurring on the XY bodies of *Dnmt3L* heterozygous males and that this ultimately leads to a three fold increased risk of XY sperm.

Sex chromosome aneuploidies are common and potentially increasing in humans. Recent data suggest that the birth incidence of Klinefelter's syndrome (XXY) is 1.09–1.75 in 1,000 [18] and 1 in 1,000 for Turner's syndrome (XO) [18], [21]. It has been shown that approximately 10% of sperm from normal healthy and fertile men have structural and numerical chromosomal abnormalities (reviewed in [22]). Sperm aneuploidies almost always arise during meiosis and defective recombination between the X and Y pseudoautosomal region is believed to be the most common cause of XY sperm disomy [23], [24]. Proposed mechanisms for their aetiology include both genetic and environmental factors. Men exposed to potential mutagens, including pesticides [25], [26] and chemotherapy agents [27], [28], have an increased risk of sperm aneuploidies. Similarly, sub-fertile and infertile men [29], [30], and mice [31], have an increased frequency of sperm chromosomal abnormalities. The data contained in this study demonstrates that decreased levels of *Dnmt3L* in germ cells could lead to a significant increase in XY sperm aneuploidy and as we have shown previously, an increased frequency of XO offspring [10].

Studies on the parental origin of the X chromosome in Turner's syndrome patients have found that in the majority of cases the remaining X chromosome is of maternal origin i.e. the paternal X or Y chromosome is lost [32]–[34]. Furthermore, a study on fathers of Turner's syndrome patients (with a single X chromosome of maternal origin) showed that these men had an increased incidence of XY sperm, suggesting an increased frequency of non-disjunction of the X and Y chromosomes during meiosis [35]. Collectively, these data raise the possibility that decreased expression of *DNMT3L* in humans may also lead to Klinefelter's or Turner's syndromes in some children.

The precise mechanisms leading to decreased XY body compaction are yet to be determined. It is possible that there are changes in the methylation status throughout the genome of *Dnmt3L* heterozygous germ cells compared to cells from wild type animals. Alternatively, or in addition, the aberrant expression of specific genes involved in spermatogenesis contributed to abnormalities. For example, *Tsga8*, which was significantly up-regulated in *Dnmt3L* heterozygous spermatocytes compared to cells from wild type males (Fig. 3A), codes for a protein involved in DNA condensation during spermatogenesis [36].

As an extension of the partial failure of XY body compaction, we anticipated that *Dnmt3L* heterozygosity would lead to an up-regulation in the transcription of X linked genes in meiotic and potentially post-meiotic germ cells. Some support for this comes from the microarray data, however, the absolute number of differentially regulated genes was small. In contrast, *Dnmt3L* heterozygosity did result in a large number of autosomal genes being differentially expressed in both spermatocytes and spermatids, revealing a previously unrecognized role of DNMT3L in the regulation of autosomal gene transcription.

*Dnmt3L* heterozygosity results in both the over- and under-expression of genes. It was anticipated that DNMT3L deficiency would predominantly be associated with increased transcription as a consequence of a looser chromatin state and increased access of transcription factors to gene promoters. While this was the case for many genes, including *Aire* and *Xrn1,* some genes, including *Ddx50* and *EPB41L2,* were significantly down-regulated. While it is clear that altered DNMT3L production results in significant changes in the expression of many genes, including *Dnmt3L* itself, it is not as yet possible to determine whether each individual change is as a direct consequence of DNMT3L deficiency, or is secondary to changes in other epigenetic regulators or transcription factors ie. these data suggest that DNMT3L sits at the start of a cascade of gene regulation networks which ultimately impact on many aspects of a cells phenotype.

Further, our microarray data show that contrary to expectations, the overall level of *Dnmt3L* transcript, based on a probe against exon 13, was unchanged. This prompted us to analyse *Dnmt3L* expression more carefully. Quantitative PCR data show that in germ cells from *Dnmt3L* heterozygous males there was up-regulated transcription of the full *Dnmt3L* splice variant from the remaining wild type allele, and there was increased use of an alternative promoter and transcription of mRNA species encoding the alternative *Dnmt3L* variants, Forms 1 and 2 in spermatids. The allele(s) from which these variants were transcribed was not analysed, however, it may have occurred from both the knockout allele and the wild type allele.

A previous study has shown that members of the DNMT3 group i.e. DNMT3L, DNMT3A and DNMT3B, can regulate methylation of the *Dnmt3L* promoter region [11]. In ES cells and embryos, a deficiency in *Dnmt3a* and *Dnmt3b* resulted in hypomethylation of the *Dnmt3L* promoter region. Similarly, a deficiency in *Dnmt3L* resulted in hypomethylation of its own promoter. The authors proposed that the DNMT3 members are involved in an auto-repression mechanism, whereby if methyltransferase activity is reduced, increased transcription of *Dnmt* family members occurs to compensate [11]. Our analysis of *Dnmt3L* expression in germ cells from wild type versus heterozygous males showed a similar mechanism exists in male meiotic and haploid germ cells. In addition, our data suggest that within at least spermatids, this mechanism includes altered transcription of the non-coding alternative *Dnmt3L* transcripts.

While not directly tested here, the simplest explanation for increased transcription of *Dnmt3L* in germ cells from heterozygous males, is hypomethylation of the CpG promoter region of *Dnmt3L*. In support of this hypothesis, we detected a dramatic increase in *Aire* expression in both spermatocytes and spermatids (Fig. 3B and C). The *Dnmt3L* and *Aire* promoters are tightly linked on chromosome 10 in the mouse, with only 6.5 kb separating the first exon of each gene in opposite transcriptional directions [37]. AIRE (Autoimmune Regulator gene) is a transcription factor expressed in antigen presenting cells in the thymus [38], and is involved in establishing and maintaining immune tolerance [39]. Recent work has shown that AIRE is also found in spermatogonia and spermatocytes [40] and that an absence of *Aire* leads to male infertility most likely at the level of the epididymis [41]. Our data shows that *Aire* is also expressed in spermatids and strongly supports a co-regulatory relationship between *Aire* and *Dnmt3L*. As both *Aire* and *Dnmt3L* promoters are known to be regulated by CpG island methylation, the compensatory up-regulation of the full length *Dnmt3L* splice variant from the remaining wild type allele, and the prominent over-expression of *Aire* in both spermatocytes and spermatids from *Dnmt3L* heterozygous males, strongly supports a mechanism whereby hypomethylation of the shared promoter has lead to increased promoter access. These data also raise the possibility that at least part of the phenotype observed herein is a consequence of increased *Aire* transcription.

Despite mRNA data showing *Dnmt3L* expression in the testis, the detection of DNMT3L in the adult testis or in sperm has not previously been reported. Our data confirmed the expression of all four *Dnmt3L* splice variants in spermatocytes and round spermatids, and we have localized DNMT3L protein in round and elongating spermatids and in mature sperm. Further, the presence of DNMT3L in wildtype sperm raise the possibility that DNMT3L is carried through to the zygote/embryo where it may impact on health. The potential role of DNMT3L in the embryo was beyond the scope of this study.

Collectively, we have shown that the loss of a *Dnmt3L* allele results in a partial failure of X chromosome condensation during male meiosis and subsequently, a three fold increase in XY chromosome non-disjunction. We have shown that altered *Dnmt3L* expression results in changes in spermatocyte and spermatid gene expression. We have shown that there is a previously unrecognised compensatory up-regulation of both the *Dnmt3L* full length and alternative transcripts in germ cells from *Dnmt3L* heterozygous males. We have shown that DNMT3L protein is found within both haploid male germ cells and mature sperm. Furthermore, these data raise the clinically important prospect that deregulated *DNMT3L* production may contribute to the aetiology of human Klinefelter's and Turner's syndromes.

## Materials and Methods

### Animals

The *Dnmt3L* knockout mice were maintained in the C57B6 background as described previously [6]. All experimentation was approved by the Monash Medical Centre (ethics approval number MMCA2007/37) and Walter and Eliza Hall Institute (WEHI) Animal Ethics Committees (AEC#s 2001.044, 2004.040, 2008.010) and conformed to the standards of the National Health and Medical Research Council (NHMRC) of Australia.

Briefly, the *Dnmt3L* knockout allele was generated using a BamHI genomic fragment containing coding exons 1–7 isolated from a PAC library (RPCI-21, http://bacpac.chori.org/femmouse21.htm) derived from a 129S6/SvEvTac mouse. The Mouse Genome Informatics database (http://www.informatics.jax.org/) showed no single nucleotide polymorphisms between 129S6/SvEvTac and C57BL/6J. The LacZ reporter gene including three stop codons and the loxP flanked PGK-neomycin cassette, were sub-cloned in-frame into coding exon 5, thus disrupting the PHD-like zinc finger domain. The targeting vector was electroporated into C57BL/6 ES cells and clones selected in G418-containing medium. Targeted ES cells were microinjected into Balb/C blastocysts to generate chimeras. Chimeric males were mated with C57BL/6 females to generate C57BL/6 offspring. The PGK-Neo cassette was removed via Cre-loxP mediated excision by breeding the mice to a cre transgenic strain on a C57BL/6 background. Specifically, the cre transgenic strain was created in BALB/cJ and was backcrossed to C57BL/6J for at least 10 generations (http://jaxmice.jax.org/strain/006054.html). The cre (and thus BALB/cJ) -ve *Dnmt3L* colony has been maintained by crossing heterozygotes or crossing to wildtype C57BL/6 for well over 10 generations. Thus the wildtype and heterozygous mice used in these experiments are essentially C57BL/6.

### Meiotic Spreads and immunofluorescent labelling

Methods for meiotic spreads were prepared as described previously [42]. Following fixation, slides were washed in 10% antibody dilution buffer (ADB) (PBS, 10% horse serum, 3% BSA, 0.05% Triton-X) three times for 15 minutes each. In order to measure XY body length, meiotic spreads were incubated with a 1/100 dilution of anti-SCP3 serum (ab15092, Abcam) and a 1/2,000 dilution of anti-phospho-Histone H2AX serum (05-636, Upstate) in 10% ADB. Slides were incubated at 37°C for 2 hours or overnight at 4°C in a dark box. Alexa Fluor secondary antibodies (Molecular Probes) were used at a 1/200 dilution in 10% ADB and applied to each section. Slides were incubated for 1 hour at 37°C or for 2 hours at room temperature in a dark box and then washed in PBS three times for 15 minutes each. Nuclear DNA was stained with DAPI and slides were mounted under coverslips with DAKO antifade mounting media.

### XY body length measurements and analysis

Immunolabelled meiotic spreads were visualized and photographed using fluorescence microscopy. Each photograph was visually assessed, then XY body lengths were traced and measured using the Image Pro Plus software program. All visual assessments and measurements were conducted without knowledge of genotype. In order to graph (but not analyse) data, measurements were grouped into 2 µm increments between 6–40 µm. To analyze the XY body length data, a general mixed linear model was used for statistical analysis using the Stata software program. This statistical analysis accounted for clustering within the mice by treating mice as random-effects and estimated differences in mean lengths between the genotypes.

### Sperm FISH for X and Y chromosome content

Methods for sperm decondensation and FISH were adapted from Whyte *et al* [43]. To enable FISH probes to easily pass into the sperm nucleus, sperm heads needed to be decondensed. Sperm were collected from dissected cauda epididymides by snipping at several locations and placed into sterile pre-warmed PBS at 37°C to allow the sperm to swim out. The sperm solution was filtered through a 100 µm mesh filter and spotted onto Superfrost plus microscope slides (Menzel-Gläser). Using the edge of a coverslip the drops were smeared along the slide as a thin film and slides were dried on a slide warmer.

The sperm smears were dehydrated in 80% methanol at −20°C for 20 minutes and air dried. A microwave safe plastic container with a divider in the middle was filled with 100 ml of distilled water and two slides were placed on the top section of the container above the water. A combination of microwaving while incubating slides in a reducing agent was conducted. 200 µl of 10 mM Dithiothreitol (DTT) was applied to the slides and the samples were covered with a coverslip. The slides were microwaved in the container for 15 seconds at 550W, then drained and 200 µl of 10 mM Lithium diiodosalicylate/1 mM DTT buffer was applied. The slides were covered with a coverslip and microwaved for 90 seconds at 550 W, then rinsed twice with 2X SSC and air dried for 30 minutes and after fixing with methanol:acetic acid (3:1) for 10 minutes, they were air dried and used immediately for FISH.

To prevent excessive chromosome denaturation, slides were placed on a slide warmer at 50°C for 1 hour. 1 µl of each of the X (directly labelled with FITC) and Y (directly labelled with CY3) probes (Cambio Ltd.) were diluted together in 3 µl of hybridization buffer (Cambio Ltd.). The probes were denatured at 65°C for 10 minutes, then pre-annealed at 37°C for 30 minutes and applied to the slides and the area was covered with a 10 mm^2^ coverslip and sealed with rubber glue. The probes, together with the sperm, were denatured by placing the slides on a slide warmer at 75°C for 3 minutes, and then incubated overnight at 37°C in a humidified chamber.

Following removal of the rubber glue, post-hybridization washes were conducted as follows: (a) 1X SSC solution at 70°C for 2–3 minutes, (b) 1X SSC/50% formamide at 45°C twice for 5 minutes each, (c) 1X SSC at 45°C twice for 5 minutes each, (d) 4X SSC/0.05% Tween 20 at 45°C twice for 5 minutes each. Nuclear DNA was stained with DAPI and slides were mounted with DAKO anti-fade mounting media.

### Analysis of sperm FISH slides

Sperm were photographed using a fluorescence microscope. The number of sperm carrying X, Y or XY chromosomes were counted without prior knowledge of the genotype. A negative binomial regression model was fitted to the data using Stata statistical software. This model determined the relative risk of sperm having X, Y or XY chromosome content, and allowed us to test for differences between wild type and heterozygous mice while allowing for heterogeneity between mice.

### Germ cell isolation

Germ cell sub-types were purified using centrifugal elutriation as described previously [44], [45]. Testes were dissected out of 10 adult mice per replicate. Briefly, tubules were removed from the tunica and chopped well and placed into a flask containing 20 ml PBS with 1 mg/ml trypsin, 1 mg/ml collagenase, 0.5 mg/ml hyaluronidase and 8 µg/ml DNase solution and shaken in a water bath at approximately 50 cycles/minute for 25 minutes. The solution was resuspended and 20 ml of PBS was added to the flask. The flask was shaken again at approximately 60 cycles/minute for 10 minutes and the solution was filtered through an 80 µm mesh and the cells were centrifuged at 430 g for 10 minutes. Cells were resuspended in 50 ml of PBS and separated by centrifugal elutriation [44], [45]. The two fractions of cells containing the round spermatids and pachytene spermatocytes were kept. Cell counts were conducted to obtain the purity of the cell fractions and only preparations with a purity of >80% were used for RNA isolation. The cells were snap-frozen in dry ice and stored at −80°C.

### RNA isolation from germ cells

For cell counts of <10,000, the Qiagen RNeasy mini kit with on column Dnase digestion was used to purify RNA. For >10,000 cells, TRIzol reagent (Invitrogen) was used. RNA extracted by the TRIzol method was Dnase treated using the Qiagen RNeasy mini kit on column Dnase digestion.

### Microarray analysis of purified germ cell gene expression

RNA from *Dnmt3L* heterozygous and wild type spermatocytes and spermatids were hybridized at the Australian Genome Research Facility Ltd (Melbourne, Australia) using their standard methods. The microarrays used in this project were Illumina Mouse Whole-Genome WG-6 Version 1.1 BeadChips. Three preparations of purified wild type spermatocytes and three preparations of purified heterozygous spermatocytes were hybridized onto one BeadChip. Similarly, three preparations of purified wild type spermatids and three preparations of purified heterozygous spermatids were hybridized onto a separate BeadChip. Each preparation of purified cells was made up of cells isolated from 10 mice. The sample probe profile data and control gene profile data were output from BeadStudio for subsequent statistical analysis in the Bioconductor software environment [46].

The *lumi* software package was used for the pre-processing and normalization. Quality control plots of intensity values showed good consistency between BeadChips and individual arrays. Probe intensities were background corrected by subtracting the mean of the negative controls for each array. A variance-stabilising transformation was applied to the corrected intensities for each array [47]. Finally the log2-expression values were quantile normalized between arrays [48]. Raw and normalized expression data and associated information is MAIME compliant and has been submitted to the GEO repository as series GSE20241. (Manuscript reviewers can access the GEO series prior to public release using the following link: http://www.ncbi.nlm.nih.gov/geo/query/acc.cgi?token=vdilhuomcocumvk&acc=GSE20241.)

Probe annotation, including gene symbol and chromosome location was obtained from the University of Cambridge (www.compbio.group.cam.ac.uk/Resources/Annotation). Negative controls on each array showed that approximately 40% of probes were not expressed in each sample. Therefore probes with the lowest 40% of mean log2-expression levels across all arrays were filtered out. This left 27,994 probes for downstream analysis.

Statistical analysis used the *limma* software package [49]. A linear model was fitted to the normalized log2-expression values for each probe. Comparisons were made between *Dnmt3L* heterozygous and wild type spermatocytes, between *Dnmt3L* heterozygous and wild type spermatids, and between the two cell types for each genotype. The standard errors were smoothed across probes using an empirical Bayes method to obtain moderated t-statistics for each comparison and each probe [50]. The false discovery rate was controlled globally across all contrasts using the method by Benjamini and Hochberg [51]. As well as the genome-wide analysis, a separate analysis was conducted using only the 855 probes on the X chromosome.

These analyses yielded log2 fold changes, average log2-expression, moderated t-statistics and false discovery rates for each probe. The comparisons were calibrated such that probes up-regulated in the *Dnmt3L* heterozygous samples relative to the wild type cells have negative log-fold changes and down-regulated probes have positive log-fold changes. In this way, fold change can be interpreted as direct responses to *Dnmt3L* dose.

### Quantitative Real-time PCR (QPCR)

For QPCR analyses, three preparations each of purified *Dnmt3L* heterozygous and wild type spermatocytes and spermatids were used. Each preparation was made up of cells isolated from 10 mice. SYBR Green primers were ordered from Sigma Aldrich and Taqman probes were ordered from Applied Biosystems. Primer sequences are listed in the Supporting Information File S1.

QPCR was performed with samples from the heterozygous and wild type males with three replicates each in a 7900 HT Fast Real Time PCR System (Applied Biosystems). SYBR Green reactions were 10 µl reactions containing 1X SYBR Green PCR Master Mix (Applied Biosystems), primer mix, and cDNA. Conditions for QPCR with SYBR included an initial step of 10 minutes at 95°C, followed by 40 thermal cycles of 15 seconds at 95°C, 30 seconds at 60°C or 62°C, 30 seconds at 72°C. At the end, samples were subjected to a slow temperature-ramping dissociation stage of 15 seconds at 95°C, 15 seconds at 60°C, 15 seconds at 95°C to monitor the specificity of amplification.

Taqman reactions were 10 µl containing 1X Taqman Universal PCR Master Mix (Applied Biosystems), Taqman probe (which included forward and reverse primers) and cDNA. QPCR conditions for QPCR with Taqman included an initial step of 10 minutes at 95°C, followed by 40 thermal cycles of 15 seconds at 95°C, 30 seconds at 60°C.

The relative standard curve method was used to quantify gene expression levels for both SYBR Green and Taqman. Samples were normalized to β-actin values. Specificity was confirmed by running QPCR products on a 1% agarose gel after each QPCR experiment. All expression values were divided by the mean of the wild type samples for that gene. One-sided t-tests on the log-expression values were used to test for differential expression, using the direction of change observed from the microarrays as the alternative hypothesis.

### Immunohistochemistry

As described previously [52]. A 1/100 dilution of the goat anti-DNMT3L (N-14) serum (Santa Cruz) and a 1/100 dilution of goat anti-DNMT3L (P-15) serum (Santa Cruz) were used separately in the immunohistochemical procedure.

### Western Blot Analysis

Sperm lysates were collected and protein extracted using 6 M urea/2% CHAPS/2% zwittergent 3–10/10 mM Tris supplemented with 5 µg/ml DNase, 5 µg/ml RNase and protease inhibitor cocktail. Western blotting was conducted as described previously [52]. A 1/1,000 dilution of mouse anti-DNMT3L serum (ME006, Shanghai Immune Biotech Co. Ltd.) was used.

For Western blot analyses, three samples each of *Dnmt3L* heterozygous and wild type sperm were used. Each sample was made up of sperm lysates isolated from 2 mice. The Kodak Imagestation 4000 MM Pro was used to quantitate the amount of DNMT3L and ACTIN within each sample. All quantity values were divided by the mean of the wild-type samples. Two-sided t-tests on these values were used to test for statistical significance.

## Supporting Information

File S1 (DOC)

## References

[1] Hammoud SS, Nix DA, Zhang H, Purwar J, Carrell DT (2009). Distinctive chromatin in human sperm packages genes for embryo development.. Nature.

[2] Houshdaran S, Cortessis VK, Siegmund K, Yang A, Laird PW (2007). Widespread epigenetic abnormalities suggest a broad DNA methylation erasure defect in abnormal human sperm.. PLoS One.

[3] Marques CJ, Costa P, Vaz B, Carvalho F, Fernandes S (2008). Abnormal methylation of imprinted genes in human sperm is associated with oligozoospermia.. Mol Hum Reprod.

[4] Bourc'his D, Bestor TH (2004). Meiotic catastrophe and retrotransposon reactivation in male germ cells lacking Dnmt3L.. Nature.

[5] La Salle S, Oakes C, Neaga O, Bourc'his D, Bestor T Loss of spermatogonia and wide-spread DNA methylation defects in newborn male mice deficient in DNMT3L.. BMC Dev Biol.

[6] Webster KE, O'Bryan MK, Fletcher S, Crewther PE, Aapola U (2005). Meiotic and epigenetic defects in Dnmt3L-knockout mouse spermatogenesis.. Proc Natl Acad Sci U S A.

[7] Sakai Y, Suetake I, Shinozaki F, Yamashina S, Tajima S (2004). Co-expression of de novo DNA methyltransferases Dnmt3a2 and Dnmt3L in gonocytes of mouse embryos.. Gene Expr Patterns.

[8] Hata K, Okano M, Lei H, Li E (2002). Dnmt3L cooperates with the Dnmt3 family of de novo DNA methyltransferases to establish maternal imprints in mice.. Development.

[9] Shovlin TC, Bourc'his D, La Salle S, O'Doherty A, Trasler JM (2007). Sex-specific promoters regulate Dnmt3L expression in mouse germ cells.. Hum Reprod.

[10] Chong S, Vickaryous N, Ashe A, Zamudio N, Youngson N (2007). Modifiers of epigenetic reprogramming show paternal effects in the mouse.. Nat Genet.

[11] Hu YG, Hirasawa R, Hu JL, Hata K, Li CL (2008). Regulation of DNA methylation activity through Dnmt3L promoter methylation by Dnmt3 enzymes in embryonic development.. Hum Mol Genet.

[12] Handel MA (2004). The XY body: a specialized meiotic chromatin domain.. Exp Cell Res.

[13] Turner JM (2007). Meiotic sex chromosome inactivation.. Development.

[14] Baarends WM, Wassenaar E, van der Laan R, Hoogerbrugge J, Sleddens-Linkels E (2005). Silencing of unpaired chromatin and histone H2A ubiquitination in mammalian meiosis.. Mol Cell Biol.

[15] van der Laan R, Uringa EJ, Wassenaar E, Hoogerbrugge JW, Sleddens E (2004). Ubiquitin ligase Rad18Sc localizes to the XY body and to other chromosomal regions that are unpaired and transcriptionally silenced during male meiotic prophase.. J Cell Sci.

[16] Tres LL (1977). Extensive pairing of the XY bivalent in mouse spermatocytes as visualized by whole-mount electron microscopy.. J Cell Sci.

[17] Paduch DA, Bolyakov A, Cohen P, Travis A (2009). Reproduction in men with Klinefelter syndrome: the past, the present, and the future.. Semin Reprod Med.

[18] Morris JK, Alberman E, Scott C, Jacobs P (2008). Is the prevalence of Klinefelter syndrome increasing?. Eur J Hum Genet.

[19] Ventela S, Toppari J, Parvinen M (2003). Intercellular organelle traffic through cytoplasmic bridges in early spermatids of the rat: mechanisms of haploid gene product sharing.. Mol Biol Cell.

[20] Mahadevaiah SK, Bourc'his D, de Rooij DG, Bestor TH, Turner JM (2008). Extensive meiotic asynapsis in mice antagonises meiotic silencing of unsynapsed chromatin and consequently disrupts meiotic sex chromosome inactivation.. J Cell Biol.

[21] Nielsen J, Wohlert M (1991). Chromosome abnormalities found among 34,910 newborn children: results from a 13-year incidence study in Arhus, Denmark.. Hum Genet.

[22] Guttenbach M, Engel W, Schmid M (1997). Analysis of structural and numerical chromosome abnormalities in sperm of normal men and carriers of constitutional chromosome aberrations. A review.. Hum Genet.

[23] Ferguson KA, Wong EC, Chow V, Nigro M, Ma S (2007). Abnormal meiotic recombination in infertile men and its association with sperm aneuploidy.. Hum Mol Genet.

[24] Hassold TJ, Sherman SL, Pettay D, Page DC, Jacobs PA (1991). XY chromosome nondisjunction in man is associated with diminished recombination in the pseudoautosomal region.. Am J Hum Genet.

[25] Padungtod C, Hassold TJ, Millie E, Ryan LM, Savitz DA (1999). Sperm aneuploidy among Chinese pesticide factory workers: scoring by the FISH method.. Am J Ind Med.

[26] Xia Y, Bian Q, Xu L, Cheng S, Song L (2004). Genotoxic effects on human spermatozoa among pesticide factory workers exposed to fenvalerate.. Toxicology.

[27] Frias S, Van Hummelen P, Meistrich ML, Lowe XR, Hagemeister FB (2003). NOVP chemotherapy for Hodgkin's disease transiently induces sperm aneuploidies associated with the major clinical aneuploidy syndromes involving chromosomes X, Y, 18, and 21.. Cancer Res.

[28] Robbins WA, Meistrich ML, Moore D, Hagemeister FB, Weier HU (1997). Chemotherapy induces transient sex chromosomal and autosomal aneuploidy in human sperm.. Nat Genet.

[29] Martin RH, Rademaker AW, Greene C, Ko E, Hoang T (2003). A comparison of the frequency of sperm chromosome abnormalities in men with mild, moderate, and severe oligozoospermia.. Biol Reprod.

[30] Templado C, Hoang T, Greene C, Rademaker A, Chernos J (2002). Aneuploid spermatozoa in infertile men: teratozoospermia.. Mol Reprod Dev.

[31] Oppedisano L, Haines G, Hrabchak C, Fimia G, Elliott R (2002). The rate of aneuploidy is altered in spermatids from infertile mice.. Hum Reprod.

[32] Cockwell A, MacKenzie M, Youings S, Jacobs P (1991). A cytogenetic and molecular study of a series of 45,X fetuses and their parents.. J Med Genet.

[33] Hassold T, Arnovitz K, Jacobs PA, May K, Robinson D (1990). The parental origin of the missing or additional chromosome in 45,X and 47,XXX females.. Birth Defects Orig Artic Ser.

[34] Jacobs P, Dalton P, James R, Mosse K, Power M (1997). Turner syndrome: a cytogenetic and molecular study.. Ann Hum Genet.

[35] Martinez-Pasarell O, Nogues C, Bosch M, Egozcue J, Templado C (1999). Analysis of sex chromosome aneuploidy in sperm from fathers of Turner syndrome patients.. Hum Genet.

[36] Uchida K, Tsuchida J, Tanaka H, Koga M, Nishina Y (2000). Cloning and characterization of a complementary deoxyribonucleic acid encoding haploid-specific alanine-rich acidic protein located on chromosome-X.. Biol Reprod.

[37] Mittaz L, Rossier C, Heino M, Peterson P, Krohn KJ (1999). Isolation and characterization of the mouse Aire gene.. Biochem Biophys Res Commun.

[38] Hubert FX, Kinkel SA, Webster KE, Cannon P, Crewther PE (2008). A specific anti-Aire antibody reveals aire expression is restricted to medullary thymic epithelial cells and not expressed in periphery.. J Immunol.

[39] Anderson MS, Venanzi ES, Klein L, Chen Z, Berzins SP (2002). Projection of an immunological self shadow within the thymus by the aire protein.. Science.

[40] Schaller CE, Wang CL, Beck-Engeser G, Goss L, Scott HS (2008). Expression of Aire and the early wave of apoptosis in spermatogenesis.. J Immunol.

[41] Hubert FX, Kinkel SA, Crewther PE, Cannon PZ, Webster KE (2009). Aire-deficient C57BL/6 mice mimicking the common human 13-base pair deletion mutation present with only a mild autoimmune phenotype.. J Immunol.

[42] Peters AH, Plug AW, van Vugt MJ, de Boer P (1997). A drying-down technique for the spreading of mammalian meiocytes from the male and female germline.. Chromosome Res.

[43] Whyte JJ, Roberts RM, Rosenfeld CS (2007). Fluorescent in situ hybridization for sex chromosome determination before and after fertilization in mice.. Theriogenology.

[44] Bellve AR, Millette CF, Bhatnagar YM, O'Brien DA (1977). Dissociation of the mouse testis and characterization of isolated spermatogenic cells.. J Histochem Cytochem.

[45] Meistrich ML, Longtin J, Brock WA, Grimes SR, Mace ML (1981). Purification of rat spermatogenic cells and preliminary biochemical analysis of these cells.. Biol Reprod.

[46] Gentleman RC, Carey VJ, Bates DM, Bolstad B, Dettling M (2004). Bioconductor: open software development for computational biology and bioinformatics.. Genome Biol.

[47] Lin SM, Du P, Huber W, Kibbe WA (2008). Model-based variance-stabilizing transformation for Illumina microarray data.. Nucleic Acids Res.

[48] Bolstad BM, Irizarry RA, Astrand M, Speed TP (2003). A comparison of normalization methods for high density oligonucleotide array data based on variance and bias.. Bioinformatics.

[49] Smyth GK, Michaud J, Scott HS (2005). Use of within-array replicate spots for assessing differential expression in microarray experiments.. Bioinformatics.

[50] Smyth GK (2004). Linear models and empirical bayes methods for assessing differential expression in microarray experiments.. Stat Appl Genet Mol Biol.

[51] Benjamini Y, Hochberg Y (1995). Controlling the false discovery rate: a practical and powerful approach to multiple testing.. Journal of the Royal Statistical Society Series B.

[52] Jamsai D, Bianco DM, Smith SJ, Merriner DJ, Ly-Huynh JD (2008). Characterization of gametogenetin 1 (GGN1) and its potential role in male fertility through the interaction with the ion channel regulator, cysteine-rich secretory protein 2 (CRISP2) in the sperm tail.. Reproduction.

