# Supplementary material for: DNMT3L Is a Regulator of X Chromosome Compaction and Post-Meiotic Gene Transcription
Source: PLoS One. 2011 Mar 31;6(3):e18276. doi: 10.1371/journal.pone.0018276 (PMC3069080; doi:10.1371/journal.pone.0018276)
Supplement: File S1 — (DOC) [file pone.0018276.s001.doc]

**Supplemental Figures**

**Figure S1. DSPs of *Dnmt3L* heterozygous and wildtype males.** No difference in DSP per testis was observed between the *Dnmt3L* heterozygous and wildtype males. Black bar = wildtype males, white bar = heterozygous males. Results are presented as means ± SEM.

**Figure S2. XY body transcriptional activity in *Dnmt3L* heterozygous and wildtype males as indicated by RNA polymerase II labelling.** The *Dnmt3L* heterozygous group had a greater percentage of cells with increased RNA polymerase II staining (measured as units fluorescence/µm^2^) when compared to the wildtype group. Black bars = wildtype males, n = 472 cells, n = 6 mice. White bars = heterozygous males, n = 556 cells, n = 6 mice.

**Supplemental Tables**

**Table S1. The incidence of sex chromosome disomy in *Dnmt3L*** heterozygous and wildtype sperm.

|  | **Wildtype**  **(n= 9 mice)** | **Heterozygous**  **(n= 8 mice)** |
| --- | --- | --- |
| **No. XY sperm** | 7 | 22 |
| **No. X or Y sperm** | 2056 | 2133 |
| **Total** | 2063 | 2155 |
| **Risk *** | .0033814 (0.34%) | .0102088 (1.02%) |

* A negative binomial regression which accounts for clustering by mouse showed a risk ratio estimate of RR=0.34 (95% CI: 0.132 to 0.867, P=0.024). Hence, the heterozygous mice have three times the risk of XY sperm when compared to the wildtype mice.

**Table S2. Significantly up- and down-regulated mRNAs in *Dnmt3L* wildtype spermatocytes compared to *Dnmt3L* heterozygous spermatocytes. ***

| **Illumina**  **Probe Id** | **Symbol** | **Chromosome** | **logFC** | **AveExpr** | **t** | **P.Value** | **FDR** |
| --- | --- | --- | --- | --- | --- | --- | --- |
| ILMN_2417556 | | No match | -2.0456 | 7.5098 | -12.7674 | 5.08E-07 | 0.0004 |
| ILMN_1226246 | LOC237296 | chr10 | -0.5311 | 7.0095 | -8.1247 | 2.10E-05 | 0.0021 |
| ILMN_2495725 | A930037J23Rik | chr2 | 0.3768 | 6.9980 | 6.8219 | 8.18E-05 | 0.0042 |
| ILMN_2707921 | Aire | chr10 | -2.9481 | 7.5825 | -5.7089 | 0.0003 | 0.0084 |
| ILMN_1242564 | Sult2a2 | chr7 | -0.4057 | 7.3673 | -5.5701 | 0.0004 | 0.0094 |
| ILMN_1225919 | Znrf4 | chr17 | -0.7812 | 12.2040 | -5.3484 | 0.0005 | 0.0110 |
| ILMN_2756632 | 9030407H20Rik | chr6 | -0.7330 | 7.0932 | -5.3383 | 0.0005 | 0.0111 |
| ILMN_2660364 | Nudt14 | chr12 | -0.3465 | 7.4820 | -4.7968 | 0.0010 | 0.0169 |
| ILMN_2682218 | 1100001I22Rik | chr3 | -0.3463 | 12.6948 | -4.7494 | 0.0011 | 0.0175 |
| ILMN_2759495 | Nkx3-1 | chr14 | 0.2645 | 6.9734 | 4.7220 | 0.0011 | 0.0180 |
| ILMN_1238541 | Ube3b | chr5 | 0.4370 | 9.8722 | 4.5837 | 0.0014 | 0.0202 |
| ILMN_2628629 | Cdh1 | chr8 | -0.3242 | 6.9567 | -4.5696 | 0.0014 | 0.0204 |
| ILMN_1249234 | LOC242681 | chr4 | -0.4951 | 9.7342 | -4.5158 | 0.0015 | 0.0214 |
| ILMN_1258920 | 2210012G02Rik | chr4 | -0.3971 | 9.9898 | -4.4998 | 0.0015 | 0.0217 |
| ILMN_2590219 | E130310N06 | chr17 | 0.2958 | 7.0313 | 4.4941 | 0.0015 | 0.0218 |
| ILMN_2619505 | Dnajc5b | chr3 | -0.7461 | 12.2720 | -4.4404 | 0.0017 | 0.0228 |
| ILMN_1225045 | 1700109H08Rik | chr5 | -0.5677 | 11.7896 | -4.4303 | 0.0017 | 0.0230 |
| ILMN_3163156 | Defb51 | chr8 | -0.2415 | 6.9913 | -4.3683 | 0.0019 | 0.0243 |
| ILMN_1241700 | 6720463J01Rik | chr9 | 0.2664 | 6.9370 | 4.3575 | 0.0019 | 0.0245 |
| ILMN_1236299 | Lyzl6 | chr11 | -0.8250 | 11.8309 | -4.3121 | 0.0020 | 0.0255 |
| ILMN_1250435 | 1700029G01Rik | chr4 | -0.5579 | 12.3292 | -4.2704 | 0.0021 | 0.0266 |
| ILMN_2622997 | Sesn3 | chr9 | 0.2822 | 7.0316 | 4.2132 | 0.0023 | 0.0280 |
| ILMN_2504368 | Spag4 | chr2 | -0.5015 | 12.3448 | -4.2019 | 0.0024 | 0.0282 |
| ILMN_1241093 | Ddost | chr4 | 0.3606 | 10.5112 | 4.1298 | 0.0026 | 0.0302 |
| ILMN_1240892 | Ppp4r1 | chr17 | 0.2464 | 7.0108 | 4.0732 | 0.0029 | 0.0319 |
| ILMN_2514674 | Xrn1 | chr9 | -0.3365 | 8.0976 | -4.0202 | 0.0031 | 0.0335 |
| ILMN_2632621 | Mapk11 | chr15 | -0.3222 | 7.2617 | -4.0151 | 0.0031 | 0.0337 |
| ILMN_1228534 | Fech | chr18 | 0.3611 | 7.1531 | 3.9630 | 0.0034 | 0.0354 |
| ILMN_1213787 | Aire | chr10 | -0.8146 | 7.0718 | -3.9580 | 0.0034 | 0.0355 |
| ILMN_2456057 | B230214O09Rik | chr6 | 0.3526 | 7.1087 | 3.9346 | 0.0035 | 0.0364 |
| ILMN_1224614 | Ddx50 | chr10 | 0.3446 | 7.1480 | 3.9086 | 0.0037 | 0.0373 |
| ILMN_1219154 | Mt2 | chr8 | -0.5438 | 8.5337 | -3.8912 | 0.0038 | 0.0379 |
| ILMN_1247460 | Opa1 | chr16 | 0.3024 | 7.0483 | 3.8874 | 0.0038 | 0.0381 |
| ILMN_2711782 | Tbc1d1 | chr5 | 0.2706 | 9.9808 | 3.8773 | 0.0038 | 0.0384 |
| ILMN_1258415 | Mat2a | chr6 | 0.2932 | 7.1395 | 3.8752 | 0.0038 | 0.0385 |
| ILMN_1248737 | 4930481A15Rik | chr19 | -0.3507 | 7.2108 | -3.8684 | 0.0039 | 0.0388 |
| ILMN_1220355 | A630084N20Rik | chr17 | 0.2322 | 6.9048 | 3.8426 | 0.0040 | 0.0397 |
| ILMN_2498592 | Zfp339 | chr2 | 0.3389 | 7.1719 | 3.7930 | 0.0044 | 0.0418 |
| ILMN_1250117 | Fnbp3 | chr2 | 0.2302 | 6.9506 | 3.7866 | 0.0044 | 0.0421 |
| ILMN_2717108 | Olfr1453 | chr19 | 0.2572 | 6.9773 | 3.7804 | 0.0044 | 0.0423 |
| ILMN_2771360 | Guk1 | chr11 | -0.4586 | 11.5782 | -3.7799 | 0.0044 | 0.0424 |
| ILMN_2650812 | Gdi3 | chr13 | 0.2993 | 7.0831 | 3.7715 | 0.0045 | 0.0427 |
| ILMN_1253576 | 4933405K21Rik | chr2 | -0.2334 | 7.4376 | -3.7422 | 0.0047 | 0.0440 |
| ILMN_2621544 | Nid2 | chr14 | 0.2187 | 7.0616 | 3.7296 | 0.0048 | 0.0447 |
| ILMN_2729198 | Fmnl2 | chr2 | 0.2816 | 7.5076 | 3.7263 | 0.0048 | 0.0448 |
| ILMN_1247861 | Cdh2 | chr18 | 0.2722 | 6.8537 | 3.7125 | 0.0049 | 0.0455 |
| ILMN_2682763 | 4930451I11Rik | chr7 | -0.9601 | 10.3407 | -3.7063 | 0.0050 | 0.0458 |
| ILMN_2696171 | D830014E11Rik | chr5 | -0.3816 | 7.1210 | -3.7041 | 0.0050 | 0.0459 |
| ILMN_1220086 | 2310016N21Rik | chr5 | -0.9985 | 8.5055 | -3.7028 | 0.0050 | 0.0459 |
| ILMN_2738284 | BC057893 | chr4 | 0.2288 | 6.8732 | 3.6635 | 0.0053 | 0.0478 |
| ILMN_1237289 | | chr11 | 0.2316 | 6.8999 | 3.6414 | 0.0055 | 0.0489 |
| ILMN_2618540 | Dnase2b | chr3 | -0.2214 | 6.8579 | -3.6265 | 0.0056 | 0.0497 |
| ILMN_2741446 | Asph | chr4 | -0.2731 | 7.0930 | -3.6206 | 0.0057 | 0.0500 |

* *Illumina Probe Id* is the Illumina gene ID; *Symbol* is the gene symbol; Chromosome is the chromosome location; *logFC* is the estimate of the log_2_-fold change; *AveExpr* is the average log_2_-expression for the mRNA over all arrays and channels; *t* is the moderated t-statistic; *P.Value* is the raw p-value; and *FDR* is the adjusted p-value or q-value, a conservative estimate of the false discovery rate when controlled globally across all contrasts. A negative log fold change value indicated the gene was up-regulated in the *Dnmt3L* heterozygous samples relative to wildtype samples. Genes with a positive value were down-regulated in the *Dnmt3L* heterozygous samples relative to wildtype samples.

**Table S3. Significantly up- and down-regulated mRNAs in *Dnmt3L* wildtype spermatids compared to heterozygous spermatids. ***

| **Illumina Probe Id** | **Symbol** | **Chromosome** | **logFC** | **AveExpr** | **t** | **P.Value** | **FDR** |
| --- | --- | --- | --- | --- | --- | --- | --- |
| ILMN_2417556 | | No match | -1.4135 | 7.5098 | -9.8637 | 4.39E-06 | 0.0010 |
| ILMN_2431189 | 4933433G15Rik | chr9 | -0.4161 | 7.8593 | -6.6246 | 0.0001 | 0.0047 |
| ILMN_2773215 | Epb4.1l4b | chr4 | 0.3114 | 7.0737 | 6.3590 | 0.0001 | 0.0055 |
| ILMN_1247527 | Pramel4 | chr4 | 0.8444 | 10.3762 | 5.9800 | 0.0002 | 0.0070 |
| ILMN_2551741 | 0610010I05Rik | chrM | 0.9886 | 9.4342 | 5.7927 | 0.0003 | 0.0079 |
| ILMN_2740372 | Pigo | chr4 | -0.4294 | 8.0712 | -5.5755 | 0.0004 | 0.0093 |
| ILMN_2669793 | Ccnd1 | chr7 | 0.4266 | 7.0120 | 5.5744 | 0.0004 | 0.0093 |
| ILMN_1228590 | 5730405M13Rik | chr5 | -0.4748 | 8.5892 | -5.2849 | 0.0005 | 0.0115 |
| ILMN_1220084 | Cables2 | chr2 | -0.4800 | 8.0211 | -5.1274 | 0.0006 | 0.0130 |
| ILMN_1236258 | Zfp523 | chr17 | -0.4954 | 8.6313 | -5.0722 | 0.0007 | 0.0135 |
| ILMN_2711329 | B930008I02Rik | chr11 | -0.4990 | 9.9443 | -5.0095 | 0.0008 | 0.0142 |
| ILMN_1218155 | Hsf2 | chr10 | 0.5467 | 7.6380 | 4.9317 | 0.0008 | 0.0151 |
| ILMN_1242564 | Sult2a2 | chr7 | -0.3159 | 7.3673 | -4.8488 | 0.0009 | 0.0162 |
| ILMN_1214876 | V1rh1 | chr13 | -0.2979 | 7.3188 | -4.8231 | 0.0010 | 0.0165 |
| ILMN_2454703 | 2900072D07Rik | chr4 | 0.4925 | 7.4051 | 4.8170 | 0.0010 | 0.0166 |
| ILMN_1224211 | Epb4.1l2 | chr10 | 0.7338 | 7.4299 | 4.8080 | 0.0010 | 0.0167 |
| ILMN_2514674 | Xrn1 | chr9 | -0.3588 | 8.0976 | -4.7919 | 0.0010 | 0.0169 |
| ILMN_1229560 | | chrM | 0.6180 | 12.9187 | 4.7190 | 0.0011 | 0.0180 |
| ILMN_2759266 | Rhpn1 | chr15 | -0.3933 | 9.3011 | -4.6095 | 0.0013 | 0.0198 |
| ILMN_2719460 | D8Ertd812e | chr8 | -0.5119 | 9.2093 | -4.5866 | 0.0014 | 0.0202 |
| ILMN_2510474 | Tro | chrX | 0.3499 | 6.9223 | 4.5747 | 0.0014 | 0.0204 |
| ILMN_2722389 | Scrib | chr15 | -0.4213 | 8.7675 | -4.5574 | 0.0014 | 0.0207 |
| ILMN_2610730 | BC025458 | No match | -0.3808 | 8.1028 | -4.5524 | 0.0014 | 0.0208 |
| ILMN_2704496 | 4930572J05Rik | chr15 | -0.6242 | 8.7125 | -4.5505 | 0.0014 | 0.0208 |
| ILMN_1227967 | LOC384258 | chr5 | -0.3505 | 7.5667 | -4.5052 | 0.0015 | 0.0216 |
| ILMN_2655112 | Pik3cd | chr4 | -0.3013 | 7.3075 | -4.4912 | 0.0016 | 0.0218 |
| ILMN_2466637 | 1200015E14Rik | chr9 | 0.4500 | 7.3703 | 4.4572 | 0.0016 | 0.0224 |
| ILMN_1246475 | 4933426G20Rik | chr15 | -0.4271 | 9.5276 | -4.4136 | 0.0017 | 0.0233 |
| ILMN_1231202 | LOC218304 | chr13 | -0.3898 | 8.9589 | -4.4115 | 0.0017 | 0.0234 |
| ILMN_1245757 | Nans | chr4 | 0.4009 | 7.2391 | 4.3865 | 0.0018 | 0.0239 |
| ILMN_1244632 | 1700072I22Rik | chr11 | -0.2612 | 7.4429 | -4.3627 | 0.0019 | 0.0244 |
| ILMN_1237430 | B930085C24Rik | chr1 | 0.3057 | 6.8835 | 4.3554 | 0.0019 | 0.0246 |
| ILMN_1240983 | D430035B07Rik | chr4 | 0.2695 | 7.1088 | 4.3384 | 0.0019 | 0.0249 |
| ILMN_1222132 | Tex2 | chr11 | 0.2612 | 10.6135 | 4.3252 | 0.0020 | 0.0252 |
| ILMN_1240767 | LOC383908 | chr3 | 0.2735 | 6.9278 | 4.2861 | 0.0021 | 0.0261 |
| ILMN_2529395 | LOC269529 | chr4 | 0.5427 | 7.9580 | 4.2648 | 0.0022 | 0.0267 |
| ILMN_1258652 | Rbbp2 | chr6 | 0.3110 | 7.1565 | 4.2573 | 0.0022 | 0.0268 |
| ILMN_1256938 | Pdgfa | chr5 | -0.3597 | 9.3859 | -4.2463 | 0.0022 | 0.0271 |
| ILMN_1221007 | Prkcn | chr17 | 0.2477 | 6.9401 | 4.2344 | 0.0023 | 0.0274 |
| ILMN_2730797 | Slc25a10 | chr11 | -0.5033 | 9.6791 | -4.2340 | 0.0023 | 0.0274 |
| ILMN_2717744 | Gga1 | chr15 | -0.4059 | 7.9158 | -4.2252 | 0.0023 | 0.0276 |
| ILMN_2722779 | Cpsf4 | chr5 | -0.3887 | 8.2831 | -4.2037 | 0.0024 | 0.0282 |
| ILMN_2537941 | LOC385935 | chr16 | -0.2785 | 7.0804 | -4.1878 | 0.0024 | 0.0287 |
| ILMN_1254417 | Iqcd | chr5 | -0.4571 | 8.9736 | -4.1849 | 0.0024 | 0.0287 |
| ILMN_2504161 | Zfyve9 | chr4 | 0.5742 | 8.1714 | 4.1784 | 0.0024 | 0.0289 |
| ILMN_1248198 | Iqca | chr1 | 0.7014 | 9.8908 | 4.1338 | 0.0026 | 0.0301 |
| ILMN_2641925 | Gm711 | chr2 | -0.3467 | 11.5609 | -4.1277 | 0.0026 | 0.0303 |
| ILMN_2523316 | Unc5b | chr10 | 0.2368 | 7.0316 | 4.1267 | 0.0026 | 0.0303 |
| ILMN_2675811 | Slc11a1 | chr1 | 0.7085 | 8.0579 | 4.0995 | 0.0027 | 0.0311 |
| ILMN_1259194 | Rhoc | chr3 | 0.1912 | 6.8390 | 4.0957 | 0.0028 | 0.0312 |
| ILMN_3163119 | AU040320 | chr4 | -0.4350 | 8.1961 | -4.0752 | 0.0028 | 0.0318 |
| ILMN_1225527 | 2700062C07Rik | chr18 | -0.4189 | 9.2926 | -4.0651 | 0.0029 | 0.0321 |
| ILMN_1247874 | BC011209 | chr12 | -0.2866 | 7.2552 | -4.0405 | 0.0030 | 0.0329 |
| ILMN_1226246 | LOC237296 | chr10 | -0.2362 | 7.0095 | -4.0396 | 0.0030 | 0.0329 |
| ILMN_2650856 | Cst11 | chr2 | -0.1866 | 7.0635 | -4.0165 | 0.0031 | 0.0337 |
| ILMN_1222315 | LOC269941 | chr7 | 0.4657 | 7.3685 | 4.0117 | 0.0031 | 0.0338 |
| ILMN_2655005 | Prkwnk1 | chr6 | 0.2839 | 8.0410 | 3.9886 | 0.0032 | 0.0345 |
| ILMN_1257223 | Kdr | chr5 | -0.4069 | 8.0567 | -3.9756 | 0.0033 | 0.0350 |
| ILMN_1232955 | A430090J22 | chr3 | -0.3209 | 7.6344 | -3.9609 | 0.0034 | 0.0354 |
| ILMN_2628281 | Scly | chr1 | -0.4202 | 8.8868 | -3.9527 | 0.0034 | 0.0357 |
| ILMN_2698430 | Bcl2l1 | chr2 | -0.4187 | 9.6150 | -3.9495 | 0.0034 | 0.0358 |
| ILMN_2473858 | Ttc19 | chr11 | 0.3509 | 8.4025 | 3.9323 | 0.0035 | 0.0364 |
| ILMN_2645276 | Tce1 | chr17 | -0.4703 | 10.3719 | -3.9069 | 0.0037 | 0.0374 |
| ILMN_2729890 | Phf1 | chr17 | -0.4768 | 8.0815 | -3.9047 | 0.0037 | 0.0375 |
| ILMN_2464553 | Tnpo2 | chr8 | -0.3584 | 8.2079 | -3.9002 | 0.0037 | 0.0376 |
| ILMN_1225532 | LOC386506 | chr15 | -0.3549 | 8.1674 | -3.8966 | 0.0037 | 0.0378 |
| ILMN_2695235 | Btbd2 | chr10 | -0.2416 | 8.0803 | -3.8951 | 0.0037 | 0.0378 |
| ILMN_2700430 | LOC381621 | chr5 | 0.4395 | 8.1326 | 3.8950 | 0.0037 | 0.0378 |
| ILMN_2734848 | Brd2 | chr17 | 0.3830 | 11.8554 | 3.8901 | 0.0038 | 0.0380 |
| ILMN_2688429 | Hnrpul1 | chr7 | -0.5362 | 8.6690 | -3.8896 | 0.0038 | 0.0380 |
| ILMN_1221995 | 2010012P19Rik | chr11 | -0.4776 | 8.2272 | -3.8896 | 0.0038 | 0.0380 |
| ILMN_1242172 | LOC384934 | chr9 | -0.2373 | 7.8333 | -3.8825 | 0.0038 | 0.0382 |
| ILMN_1225638 | Lnk | chr5 | -0.4520 | 7.8666 | -3.8775 | 0.0038 | 0.0384 |
| ILMN_1218523 | 5730592D20Rik | chr8 | 0.2786 | 7.0583 | 3.8761 | 0.0038 | 0.0385 |
| ILMN_1227125 | 4932442L11Rik | chr5 | -0.4470 | 8.7375 | -3.8751 | 0.0038 | 0.0385 |
| ILMN_2772288 | Disp1 | chr1_random | 0.2005 | 7.1053 | 3.8742 | 0.0039 | 0.0385 |
| ILMN_2537305 | LOC383107 | chr16 | 0.2310 | 6.9791 | 3.8677 | 0.0039 | 0.0388 |
| ILMN_2730410 | 2310075G12Rik | chr11 | -0.5046 | 10.6140 | -3.8658 | 0.0039 | 0.0389 |
| ILMN_1237935 | E030040P03Rik | chr8 | -0.2974 | 7.4410 | -3.8643 | 0.0039 | 0.0389 |
| ILMN_2566052 | C130020C07Rik | chr5 | 0.2929 | 7.1144 | 3.8580 | 0.0039 | 0.0392 |
| ILMN_1239432 | Pcqap | chr16 | -0.5031 | 8.8486 | -3.8541 | 0.0040 | 0.0393 |
| ILMN_2684455 | Tada3l | chr6 | -0.4024 | 9.5558 | -3.8523 | 0.0040 | 0.0394 |
| ILMN_1256270 | Ranbp2 | chr10 | 0.1756 | 6.8555 | 3.8478 | 0.0040 | 0.0395 |
| ILMN_1253614 | E2f2 | chr4 | 0.3949 | 7.3126 | 3.8450 | 0.0040 | 0.0397 |
| ILMN_1232052 | A730008I18Rik | chr4 | 0.3147 | 7.2369 | 3.8207 | 0.0042 | 0.0406 |
| ILMN_2728889 | Sdk1 | chr5 | 0.2346 | 7.0173 | 3.8186 | 0.0042 | 0.0407 |
| ILMN_2517450 | 2900076A13Rik | chr2 | 0.3435 | 6.9086 | 3.8057 | 0.0043 | 0.0412 |
| ILMN_2527130 | LOC329750 | chr13 | 0.2914 | 7.1323 | 3.7954 | 0.0043 | 0.0417 |
| ILMN_2525338 | BC013491 | chr7 | -0.3554 | 8.3732 | -3.7876 | 0.0044 | 0.0420 |
| ILMN_2685120 | Dpep3 | chr8 | -0.2215 | 11.8062 | -3.7859 | 0.0044 | 0.0421 |
| ILMN_1222157 | Cul1 | chr6 | 0.2653 | 8.1850 | 3.7734 | 0.0045 | 0.0426 |
| ILMN_2710229 | Ccng1 | chr11 | 0.4125 | 7.6490 | 3.7681 | 0.0045 | 0.0428 |
| ILMN_1223689 | Sf3a2 | chr10 | -0.5121 | 7.8008 | -3.7660 | 0.0045 | 0.0430 |
| ILMN_2576381 | C130088C22Rik | chr5 | -0.2279 | 7.4296 | -3.7645 | 0.0046 | 0.0430 |
| ILMN_1253196 | BC029716 | chr2 | -0.2149 | 7.2114 | -3.7599 | 0.0046 | 0.0432 |
| ILMN_1249482 | LOC210196 | chr6 | -0.2773 | 7.3533 | -3.7565 | 0.0046 | 0.0434 |
| ILMN_1251429 | 9430013L17Rik | chr10 | 0.2852 | 7.0890 | 3.7544 | 0.0046 | 0.0434 |
| ILMN_1223654 | LOC277973 | chr8 | -0.3867 | 7.7883 | -3.7383 | 0.0047 | 0.0442 |
| ILMN_2617449 | 2310047N01Rik | chr4 | -0.2361 | 6.8068 | -3.7377 | 0.0047 | 0.0442 |
| ILMN_1236534 |  | chr15 | 0.2778 | 6.9476 | 3.7350 | 0.0048 | 0.0444 |
| ILMN_1252719 | Wbscr22 | chr5 | -0.4543 | 8.6919 | -3.7270 | 0.0048 | 0.0448 |
| ILMN_1215176 | LOC383942 | chr10 | 0.2718 | 7.0113 | 3.7186 | 0.0049 | 0.0452 |
| ILMN_2597034 | Rhcg | chr7 | -0.4447 | 9.0227 | -3.7158 | 0.0049 | 0.0453 |
| ILMN_2678576 | Numb | chr12 | -0.4168 | 8.0588 | -3.7060 | 0.0050 | 0.0458 |
| ILMN_1244324 | Hdgf | chr3 | -0.3835 | 11.5006 | -3.7030 | 0.0050 | 0.0459 |
| ILMN_1243690 | A130029H05Rik | chr10 | -0.4273 | 9.9184 | -3.6914 | 0.0051 | 0.0464 |
| ILMN_2469449 | 4933404K08Rik | chr13 | -0.4508 | 8.1997 | -3.6894 | 0.0051 | 0.0465 |
| ILMN_1252338 | LOC381019 | chr15 | -0.4086 | 8.2626 | -3.6836 | 0.0052 | 0.0468 |
| ILMN_1225075 | LOC384842 | chr8 | -0.4732 | 9.2535 | -3.6832 | 0.0052 | 0.0468 |
| ILMN_1251081 | 4933424M23Rik | chr11 | -0.4775 | 8.5643 | -3.6830 | 0.0052 | 0.0468 |
| ILMN_1249164 | Dffa | chr4 | -0.3703 | 8.0336 | -3.6789 | 0.0052 | 0.0470 |
| ILMN_2452717 | 2610019E17Rik | chr17 | 0.5539 | 7.8081 | 3.6680 | 0.0053 | 0.0476 |
| ILMN_2710382 | Grin2d | chr7 | -0.1521 | 6.9221 | -3.6598 | 0.0053 | 0.0480 |
| ILMN_2675152 | 5730409F24Rik | chr19 | -0.4861 | 8.9634 | -3.6476 | 0.0054 | 0.0486 |
| ILMN_2702233 | Ccng1 | chr11 | 0.3294 | 7.1803 | 3.6470 | 0.0055 | 0.0487 |
| ILMN_2532423 | LOC243965 | chr7 | -0.4816 | 9.0842 | -3.6454 | 0.0055 | 0.0487 |
| ILMN_1245449 | Hist1h2ak | chr13 | 0.7855 | 9.6351 | 3.6448 | 0.0055 | 0.0488 |
| ILMN_1222228 | Adprt1 | chr1 | -0.5341 | 8.7613 | -3.6370 | 0.0055 | 0.0491 |
| ILMN_2621433 | Fgfrl1 | chr5 | -0.3362 | 8.2987 | -3.6355 | 0.0056 | 0.0492 |
| ILMN_2695747 | Mcm4 | chr16 | 0.6381 | 8.7773 | 3.6309 | 0.0056 | 0.0495 |
| ILMN_2742557 | Bcl2l1 | chr2 | -0.5495 | 8.6754 | -3.6298 | 0.0056 | 0.0495 |
| ILMN_1233424 | Lbcl1 | chr3 | 0.7574 | 7.9402 | 3.6249 | 0.0056 | 0.0498 |
| ILMN_1240039 | Egf | chr3 | -0.3665 | 8.4756 | -3.6231 | 0.0057 | 0.0498 |

* *Illumina Probe Id* is the Illumina gene ID; *Symbol* is the gene symbol; Chromosome is the chromosome location; *logFC* is the estimate of the log_2_-fold change; *AveExpr* is the average log_2_-expression for the mRNA over all arrays and channels; *t* is the moderated t-statistic; *P.Value* is the raw p-value; and *FDR* is the adjusted p-value or q-value, a conservative estimate of the false discovery rate when controlled globally across all contrasts. A negative log fold change value indicated the gene was up-regulated in the *Dnmt3L* heterozygous samples relative to wildtype samples. Genes with a positive value were down-regulated in the *Dnmt3L* heterozygous samples relative to wildtype samples.

**Table S4. The ten most significant differentially expressed X chromosome genes between *Dnmt3L* wildtype and heterozygous spermatocytes.***

| **Illumina Probe Id** | **Symbol** | **logFC** | **AveExpr** | **t** | **P.Value** | **FDR** |
| --- | --- | --- | --- | --- | --- | --- |
| ILMN_2438267 | Tsga8 | -0.429 | 12.654 | -3.487 | 0.007 | 0.046 |
| ILMN_1225230 | Sybl1 | -0.179 | 6.841 | -2.944 | 0.017 | 0.090 |
| ILMN_1230457 | Mic2l1 | 0.175 | 6.872 | 2.769 | 0.022 | 0.115 |
| ILMN_1239385 | D330023I21Rik | -0.177 | 7.222 | -2.693 | 0.025 | 0.126 |
| ILMN_1238634 | 4933402E13Rik | 0.254 | 7.082 | 2.650 | 0.027 | 0.132 |
| ILMN_2477690 | | -0.173 | 6.958 | -2.627 | 0.028 | 0.135 |
| ILMN_1252122 | LOC382210 | 0.259 | 7.521 | 2.605 | 0.029 | 0.139 |
| ILMN_3163371 | 4930567H17Rik | -0.193 | 6.973 | -2.599 | 0.029 | 0.140 |
| ILMN_1250043 | LOC208414 | 0.164 | 6.913 | 2.499 | 0.034 | 0.158 |
| ILMN_1226793 | Gm1549 | -0.153 | 6.994 | -2.464 | 0.036 | 0.165 |
| ILMN_1233065 | Rbmx | 0.14 | 6.98 | 2.37 | 0.04235 | 0.1855 |
| ILMN_3162365 | A130028J20Rik | -0.129 | 6.97 | -2.34 | 0.04468 | 0.1927 |

* *Illumina Probe Id* is the Illumina gene ID; *Symbol* is the gene symbol; Chromosome is the chromosome location; *logFC* is the estimate of the log_2_-fold change; *AveExpr* is the average log_2_-expression for the mRNA over all arrays and channels; *t* is the moderated t-statistic; *P.Value* is the raw p-value; and *FDR* is the adjusted p-value or q-value, a conservative estimate of the false discovery rate when controlled globally across all contrasts. A negative log fold change value indicated the gene was up-regulated in the *Dnmt3L* heterozygous samples relative to wildtype samples. Genes with a positive value were down-regulated in the *Dnmt3L* heterozygous samples relative to wildtype samples.

**Table S5. The most significant differentially expressed X chromosome genes between *Dnmt3L* wildtype and heterozygous spermatids.***

| **Illumina**  **Probe Id** | **Symbol** | **logFC** | **AveExpr** | **t** | **P.Value** | **FDR** |
| --- | --- | --- | --- | --- | --- | --- |
| ILMN_2510474 | Tro | 0.3499 | 6.9223 | 4.5747 | 0.0014 | 0.0141 |
| ILMN_2420366 | U2af1-rs2 | 0.4690 | 7.7304 | 3.4835 | 0.0070 | 0.0458 |
| ILMN_1212894 | Cask | 0.3169 | 7.0874 | 3.4604 | 0.0073 | 0.0469 |
| ILMN_2447456 | Utx | 0.4051 | 7.6106 | 3.4559 | 0.0073 | 0.0471 |
| ILMN_2432018 | 2900057E15Rik | 0.2345 | 6.9084 | 3.4551 | 0.0073 | 0.0471 |
| ILMN_1247127 | B930075F07 | 0.3006 | 7.0441 | 3.4308 | 0.0076 | 0.0483 |
| ILMN_1250353 | LOC236427 | 0.4037 | 7.8292 | 3.4231 | 0.0077 | 0.0487 |
| ILMN_2506528 | Utx | 0.3548 | 7.6350 | 3.3663 | 0.0085 | 0.0524 |
| ILMN_2611676 | Ogt | 0.2171 | 6.8058 | 3.2987 | 0.0094 | 0.0570 |
| ILMN_1249012 | 4930524N10Rik | 0.7048 | 9.4720 | 3.2562 | 0.0101 | 0.0601 |
| ILMN_2615810 | Nudt11 | 0.323 | 7.2 | 3.2 | 0.01098 | 0.0645 |
| ILMN_1253181 | LOC277860 | -0.345 | 8.64 | -3.1 | 0.01288 | 0.0736 |
| ILMN_1257565 | LOC331416 | 0.408 | 7.28 | 3.06 | 0.01385 | 0.0779 |
| ILMN_2704499 | Hprt | 0.524 | 8.42 | 3.04 | 0.0142 | 0.0795 |
| ILMN_1228320 | Pfc | 0.694 | 7.54 | 3.03 | 0.0144 | 0.0805 |
| ILMN_1229689 | Ammecr1 | 0.264 | 6.92 | 2.99 | 0.01545 | 0.0852 |
| ILMN_1217489 | Sms | 0.357 | 7.47 | 2.96 | 0.01627 | 0.089 |
| ILMN_2674890 | Tbl1x | 0.341 | 7.44 | 2.93 | 0.017 | 0.0921 |
| ILMN_2437848 | Col4a5 | 0.457 | 6.95 | 2.92 | 0.01726 | 0.0932 |
| ILMN_2698589 | Cstf2 | 0.451 | 7.73 | 2.87 | 0.01875 | 0.1002 |
| ILMN_2562032 | A130070G01Rik | 0.342 | 7.43 | 2.73 | 0.02335 | 0.1202 |
| ILMN_1220292 | Gla | -0.299 | 7.61 | -2.73 | 0.02357 | 0.1207 |
| ILMN_2684324 | Dock11 | 0.287 | 7.11 | 2.69 | 0.02504 | 0.1259 |
| ILMN_2420209 | 6430511F03 | 0.533 | 7.08 | 2.69 | 0.02517 | 0.1264 |
| ILMN_2592886 | Bhlhb9 | 0.401 | 7.8 | 2.68 | 0.02566 | 0.128 |
| ILMN_1260307 | Piga | 0.176 | 6.87 | 2.67 | 0.02581 | 0.1285 |
| ILMN_3162365 | A130028J20Rik | -0.129 | 6.97 | -2.62 | 0.02819 | 0.1368 |
| ILMN_1249209 | C530025M17Rik | 0.152 | 6.94 | 2.61 | 0.02859 | 0.1385 |
| ILMN_2631828 | BC004701 | -0.152 | 6.92 | -2.59 | 0.02934 | 0.1404 |
| ILMN_2455089 | Xlr4 | 0.147 | 6.88 | 2.59 | 0.02965 | 0.1414 |
| ILMN_2599470 | Tex11 | 0.363 | 7.19 | 2.58 | 0.02994 | 0.1426 |
| ILMN_2429250 | Rnu70 | 0.118 | 6.92 | 2.56 | 0.03112 | 0.1472 |
| ILMN_1247078 | Ogt | 0.217 | 7.19 | 2.55 | 0.03124 | 0.1474 |
| ILMN_1232093 | LOC386330 | 0.621 | 7.8 | 2.53 | 0.0323 | 0.152 |
| ILMN_3161155 | A830080D01Rik | -0.243 | 7.09 | -2.53 | 0.03248 | 0.1526 |
| ILMN_1248608 | Apxl | 0.336 | 7.25 | 2.52 | 0.03289 | 0.1543 |
| ILMN_1258525 | 4930570D08Rik | -0.181 | 7.45 | -2.51 | 0.03343 | 0.1558 |
| ILMN_1231277 | LOC382231 | 0.396 | 8.06 | 2.51 | 0.03359 | 0.1563 |
| ILMN_2759749 | Syap1 | 0.171 | 7.04 | 2.5 | 0.03401 | 0.1576 |
| ILMN_1247896 | LOC279618 | 0.124 | 6.85 | 2.47 | 0.03576 | 0.1635 |
| ILMN_2711551 | Rpl39 | 0.909 | 8.16 | 2.47 | 0.03614 | 0.1646 |
| ILMN_1247009 | Rps6ka3 | 0.148 | 6.87 | 2.44 | 0.03773 | 0.1702 |
| ILMN_1233065 | Rbmx | 0.127 | 6.98 | 2.41 | 0.03986 | 0.178 |
| ILMN_1249467 | Rap2c | 0.291 | 7.21 | 2.39 | 0.04096 | 0.1819 |
| ILMN_2599532 | Gdi1 | 0.447 | 7.23 | 2.38 | 0.04123 | 0.1826 |
| ILMN_2622041 | F8a | 0.142 | 7.09 | 2.38 | 0.04127 | 0.1826 |
| ILMN_1235027 | Col4a6 | 0.262 | 6.95 | 2.38 | 0.04135 | 0.1827 |
| ILMN_2689773 | BC022692 | -0.198 | 7.02 | -2.36 | 0.04262 | 0.1862 |
| ILMN_1216083 | Araf | 0.188 | 7.06 | 2.36 | 0.04271 | 0.1863 |
| ILMN_1237603 | LOC331381 | 0.185 | 6.92 | 2.36 | 0.04319 | 0.1875 |
| ILMN_1219646 | Rpl36a | 0.932 | 9.18 | 2.36 | 0.04326 | 0.1875 |
| ILMN_2429302 | A730046J19Rik | -0.163 | 6.89 | -2.35 | 0.0439 | 0.1898 |
| ILMN_2540151 | LOC385378 | -0.161 | 7.24 | -2.33 | 0.04527 | 0.1942 |
| ILMN_2762755 | 1500005J14Rik | -0.123 | 6.81 | -2.32 | 0.04608 | 0.1972 |

* *Illumina Probe Id* is the Illumina gene ID; *Symbol* is the gene symbol; Chromosome is the chromosome location; *logFC* is the estimate of the log_2_-fold change; *AveExpr* is the average log_2_-expression for the mRNA over all arrays and channels; *t* is the moderated t-statistic; *P.Value* is the raw p-value; and *FDR* is the adjusted p-value or q-value, a conservative estimate of the false discovery rate when controlled globally across all contrasts. A negative log fold change value indicated the gene was up-regulated in the *Dnmt3L* heterozygous samples relative to wildtype samples. Genes with a positive value were down-regulated in the *Dnmt3L* heterozygous samples relative to wildtype samples.

Table S6. Details of primers used in QPCR experiments.

| **primer name** | **gene target** | **sequence: 5’ – 3’** |
| --- | --- | --- |
| Bactin-F | Bactin, control | GATCTGGCACCACACCTTCT |
| Bactin-R | Bactin, control | GGGGTGTTGAAGGTCTCAAA |
| Tsga8-F | Tsga8, X-linked spermatocyte | CCTGGTGACGAACCAGACTT |
| Tsga-R | Tsga8, X-linked spermatocyte | GCTTCACCTCCTGAGAATCG |
| H17-F | H17Rik, X-linked spermatocyte | GGGCCTCTGAGACCACATAA |
| H17-R | H17Rik, X-linked spermatocyte | CGCTCATACCCAGGTACGTC |
| Sybl-F | Sybl1, X-linked spermatocyte | TTTTGGCTGCACAACTGAAG |
| Sybl-R | Sybl1, X-linked spermatocyte | GCAATTCCAACCTTTCTCCA |
| LOC-F | LOC208414, X-linked spermatocyte | GAACGGCATAACCCAACAGT |
| LOC-R | LOC208414, X-linked  spermatocyte | CGTCTGCCTCTTCCAAAGTC |
| Cd9912-F | Cd9912, X-linked spermatocyte | GTCCAGGTCTGAGGATCCAA |
| Cd9912-R | Cd9912, X-linked spermatocyte | TGCAAGTGATGCCTTCTTTG |
| E13-F | E13Rik, X-linked spermatocyte | CTTCCAGAATCCAACGGAAA |
| E13-R | E13Rik, X-linked spermatocyte | TCGTCTTCCTTCTCGACGTT |
| Xrn1-F | Xrn1, autosomal spermatocyte and spermatid | GGCGTGAGTGGATACCTTGT |
| Xrn1-R | Xrn1, autosomal spermatocyte and spermatid | AGCCAGGGACTTCCTCATTT |
| DNAjc-F | DNAjc5b, autosomal spermatocyte | CACACAATCCTCACCGACAC |
| DNAjc-R | DNAjc5b, autosomal spermatocyte | ATGATGAACAGGGTCCTTGC |
| Spag-F | Spag4, autosomal spermatocyte | CCTACTTCTGGAACCGCTTG |
| Spag-R | Spag4, autosomal spermatocyte | CCTGGCCTTTATCACCTTCA |
| Ddx-F | Ddx50, autosomal spermatocyte | TGGGTCTGAAGGGAGAGCTA |
| Ddx-R | Ddx50, autosomal spermatocyte | TCTCCATGCAAACACTGAGC |
| Fech-F | Fech, autosomal spermatocyte | GCGAGGTGGTCATTCTGTTT |
| Fech-R | Fech, autosomal spermatocyte | ACTGGACCAACCTTGGACTG |
| Ddost-F | Ddost, autosomal spermatocyte | AACTAGCTGTGGCCCTCTCA |
| Ddost-R | Ddost, autosomal spermatocyte | TGGAAGATTTTGGAGGCAAC |
| Aire-F | Aire, autosomal spermatocyte and spermatid | GGGACGACTCTGCTAGTCAC |
| Aire-R | Aire, autosomal spermatocyte and spermatid | CATCCTGCAGTGGGCCATCC |
| Bcl-F | Bcl2l1, autosomal spermatid | TTCGGGATGGAGTAAACTGG |
| Bcl-R | Bcl2l1, autosomal spermatid | TGCAATCCGACTCACCAATA |
| Parp-F | Parp1, autosomal spermatid | CACCCTCCAAGAAGAGCAAG |
| Parp-R | Parp1, autosomal spermatid | TGCAGAGTGTTCCAGACCAG |
| Slc-F | Slc11a1, autosomal spermatid | AACATCTGTGCCAACAGCAG |
| Slc-R | Slc11a1, autosomal spermatid | GCATAGGTGCCGGTCATAGT |
| EPB-F | EPB41L2, autosomal spermatid | CCTATTGTAGCCCCTCGTCA |
| EPB-R | EPB41L2, autosomal spermatid | TCTTCATCAAGGAGGGATGG |
| Mcm-F | Mcm4, autosomal spermatid | GACCCTCGTACTGGCATTGT |
| Mcm-R | Mcm4, autosomal spermatid | GTGTCAGACTGTCCCCGAAT |
| Dnmt3L5-F | Dnmt3L-5 | GAACCGACGGAGCATTGAAG |
| Dnmt3L5-R | Dnmt3L-5 | CGGCACCCCTTGTTTGAGGGA |
| 3Lmid-F | Dnmt3L-mid | TCTCACGGAGTGGACTGCT |
| 3Lmid-R | Dnmt3L-mid | ACTGGCTGTCTCTTCCATGC |
| Dnmt3L3-F | Dnmt3L-3 | AGTATCTGCAAGCCCAAGTC |
| Dnmt3L3-R | Dnmt3L-3 | TGGTGAAGAACTGCCTTCTC |
